# Some generalized recurrence relations for nonlinear equations by using decomposition technique with application of fractal geometry

**DOI:** 10.1016/j.mex.2026.103835

**Published:** 2026-02-18

**Authors:** Farooq Ahmed Shah, Syeda Rameesha Hamdani, Fikadu Tesgera Tolasa, Iftikhar Haider

**Affiliations:** a Department of Mathematics, COMSATS University Islamabad, Attock Campus, Pakistan; b Department of Physics, COMSATS University Islamabad, Pakistan; c Department of Mathematics, Dambi Dollo University, Oromia, Ethiopia

**Keywords:** Iterative method, Quadrature rule, Taylor series, Decomposition method, Newton method, Convergence, Fractals

## Abstract

Nonlinear equations frequently appear in diverse fields of applied sciences, where real-world phenomena cannot be accurately represented by linear models. Therefore, developing efficient numerical methods to approximate the roots of such equations remain a challenging and intellectually stimulating task. These methods are crucial in physics, engineering and computer science for solving nonlinear equations. In response to the growing demands of real-time systems, complicated simulations and high-performance computing, this article introduces few novel root-finding methods that significantly improve the convergence order of the traditional approaches. Accelerated decomposition technique is to diversify different classes of iterative methods. Newly derived methods are compared with existing methods numerically as well as graphically. Polynomiography is employed to visualize the basins of attraction, providing insight into the convergence behavior and stability of the methods. The results indicate that the new algorithms not only overcome the limitations of existing techniques but also offer a visually intuitive understanding of root-finding processes.

This study presents innovative root-finding methods that utilize accelerated decomposition techniques.

The proposed methods demonstrate a significant improvement in convergence order compared to traditional approaches

Through numerical and graphical comparisons, the newly derived methods are shown to outperform existing methods.

$$=\;x_{n}-\frac{f\left(x_{n}\right)p\left(x_{n}\right)}{p^{\prime }\left(x_{n}\right)f\left(x_{n}\right)+f^{\prime }\left(x_{n}\right)p\left(x_{n}\right)}$$

## Graphical abstract

**Figure:**
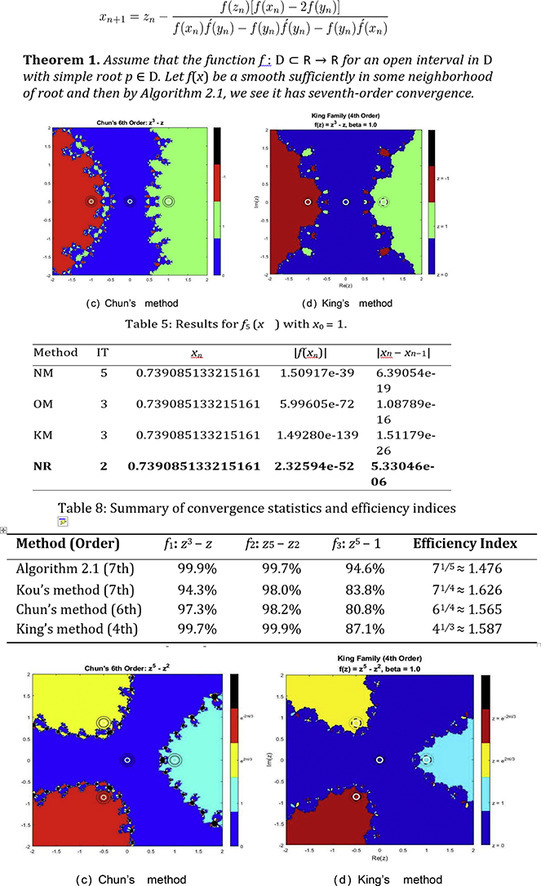

**Algorithm 2.1.** For a given $x_{0}$, compute the approximate solution $x_{n+1}$ by the following iterative scheme.

Alt-text: Unlabelled box dummy alt text

**Figure:**
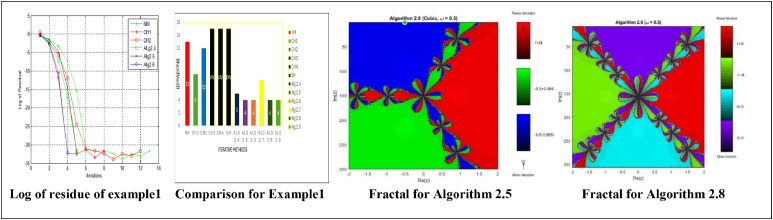

Theorem 3.1**.** Let α∈I be a simple zero of sufficiently differentiable function f:I⊆R→R for an open interval I. If $x_{0\;}$is sufficiently close to α, then iterative method define in Algorithm 2.3 has at least fourth-order convergence.

Alt-text: Unlabelled box dummy alt text

## Specifications table

**Table:** 

**Subject area**	Mathematics and Statistics
**More specific subject area**	*Numerical Analysis*
**Name of your method**	*Iterative methods for nonlinear equation*
**Name and reference of original method**	*All are newly developed methods*
**Resource availability**	*Maple is used for calculation*

## Background

Finding approximate solutions of nonlinear equations is a crucial aspect of various branches of pure and applied mathematics, playing a vital role in numerous fields. In physics, it's essential for modeling population growth, electrical circuits, mechanical systems, and quantum mechanics. Engineering relies on it for designing electronic circuits, control systems, signal processing and structural analysis. Computer science utilizes nonlinear equations in machine learning, data analysis, algorithm development and computer graphics. Biology, economics, environmental science, medicine, optimization, signal processing, chemistry, aerospace, materials science, neuroscience and finance also heavily depend on solving nonlinear equations to make predictions, model real-world phenomena and optimize systems. The significance of nonlinear equations is evident in understanding market dynamics, resource allocation, and economic growth, as well as in analyzing medical imaging, modeling disease spread and understanding pharmacokinetics. The ability to solve these equations efficiently and accurately is critical in advancing research, driving innovation and solving complicated problems across these diverse disciplines. In physics, nonlinear equations appear in quantum mechanics, nonlinear optics, plasma physics, and thermodynamics. Problems such as determining energy levels, solving dispersion relations, or computing steady states of nonlinear dynamical systems rely heavily on iterative root-finding techniques. High-order iterative methods are particularly valuable in these contexts, as they can achieve rapid convergence and high precision, reducing computational cost in large-scale simulations.

The Newton method, a widely used and powerful technique for finding roots of nonlinear equations, boasts quadratic convergence but is not without its limitations. Despite its rapid convergence, the Newton method can be susceptible to numerical instabilities, including division by zero, poor initial guesses, non-convergence and numerical overflow. To mitigate these issues, various modifications and alternatives have been developed, such as the secant method, bisection method, quasi-Newton methods, trust region methods, and hybrid methods. These refinements aim to improve the robustness and reliability of root-finding method making them more suitable for a broad range of applications. By addressing the shortcomings of the Newton method, researchers and practitioners can harness the strengths of these techniques to efficiently and accurately solve complex nonlinear equations. This method can suffer from numerical instabilities due to $f^{\prime }\left(x\right)=0$ or $f^{\prime }\left(x\right)$ be very small at any $x_{n}$ during computational process at any step. Several iterative methods have been developed for solving f(x)=0using different techniques [[1], [2], [3], [4], [5], [6], [7], [8], [9], [10], [11], [12], [13], [14], [15], [16], [17], [18], [19], [20], [21], [22], [23], [24], [25], [26], [27], [28], [29], [30], [31], [32], [33], [34], [35], [36], [37]]. Abbasbandy [1] and Chun [[8], [9], [10]] worked out to gain different one-step and two-step iterative methods by using Adomian decomposition techniques [1]. These methods are of higher order of convergence but persevere with some drawbacks. Our goal is to develop a generalized recurrence relation that can be implemented to generate iterative methods for finding more accurate solutions for nonlinear equations. In this work, we will implement the technique of Gejji & Jafari [11] to decompose the nonlinear equation for obtaining an applicable formula for nonlinear equations. We will use auxiliary functions to diversify the techniques for best implementation of the methods. We will apply quadrature for this procedure then finally we will generalize the decomposition technique of [11] which is different than Adomian decomposition [1] to formulate the higher order convergent iterative methods. The use of arbitrary and auxiliary function will make the decomposition much more flexible and applicable for derivation and implementation of the iterative methods. Convergence analysis of the newly derived iterative is also studied. Performance of these new schemes is illustrated by several examples and comparison with other methods is shown graphically.

## Method details

In this section, we develop a novel class of iterative methods for solving nonlinear equations by integrating the Taylor series expansion and numerical quadrature techniques. The Taylor series approach provides a local polynomial approximation of the nonlinear function and its derivatives, enabling the construction of higher-order iterative schemes with predictable convergence behavior. Complementing this, the quadrature-based approach utilizes the integral form of the nonlinear equation and approximates the integral using Newton–Cotes or Gaussian quadrature formulas, which effectively capture the cumulative behavior of the derivative over the iteration interval. By combining these two strategies, we construct hybrid iterative methods in which the Taylor expansion offers accurate local predictions while the quadrature evaluation refines the update step by incorporating derivative information at multiple nodes. This unified framework results in efficient, high-order Newton-type methods that maintain rapid convergence with improved stability, providing a flexible and systematic approach for generating powerful iterative algorithms for nonlinear problems. Newton method, Halley method and Householder methods are easily obtained by using Taylor series technique and quadrature rules with fundamental Theorem of callus combination provides various applicable methods. This section introduces fundamental and unique recurrence relations and with the help of these new relations innovative iterative methods for addressing nonlinear equations. By combining a system of coupled equations with decomposition techniques, we develop a robust approach to obtaining approximate solutions. The incorporation of an auxiliary function expands the versatility of the main recurrence relation, facilitating its application to a broader range of nonlinear equations.

### Consider the nonlinear equation

(1) f(x)=0.

On the condition that α as a simple root of nonlinear Eq. (1) and γ is the initial estimate which is close to α. Let g(x) be the involved auxiliary and arbitrary function, such that

(2) f(x)p(x)=0.

Using Taylor series on p(x)and quadrature formula on f(x) then Eq. (2) can be written as

(3) $$f\left(x\right)p\left(x\right)=f\left(\gamma \right)p\left(\gamma \right)+\left(x-\gamma \right)\left[p^{\prime }\left(\gamma \right)f\left(\gamma \right)+\left\{\frac{f^{\prime }\left(\gamma \right)+2f^{\prime }\left(\frac{\gamma +x}{2}\right)+f^{\prime }\left(x\right)}{4}\right\}p\left(\gamma \right)\right].$$

We can express Eq. (3) as:

(4) $$h\left(x\right)=f\left(x\right)p\left(\gamma \right)-f\left(\gamma \right)p\left(\gamma \right)-\left(x-\gamma \right)\left[p^{\prime }\left(\gamma \right)f\left(\gamma \right)+\left\{\frac{f^{\prime }\left(\gamma \right)+2f^{\prime }\left(\frac{\gamma +x}{2}\right)+f^{\prime }\left(x\right)}{4}\right\}p\left(\gamma \right)\right].$$

Eq. (4) can take the following form by simple manipulation

(5) $$x=\gamma -\frac{4\left(h\left(x\right)-f\left(x\right)p\left(\gamma \right)+f\left(\gamma \right)p\left(\gamma \right)\right)}{4p^{\prime }\left(\gamma \right)f\left(\gamma \right)+\left\{f^{\prime }\left(\gamma \right)+2f^{\prime }\left(\frac{\gamma +x}{2}\right)+f^{\prime }\left(x\right)\right\}p\left(\gamma \right)}=c+N\left(x\right).$$

Where

(6) c=γ,

and

(7) $$N\left(x\right)=-\frac{4\left(h\left(x\right)+f\left(\gamma \right)p\left(\gamma \right)\right)}{4p^{\prime }\left(\gamma \right)f\left(\gamma \right)+\left\{f^{\prime }\left(\gamma \right)+2f^{\prime }\left(\frac{\gamma +x}{2}\right)+f^{\prime }\left(x\right)\right\}p\left(\gamma \right)}.$$

Where N(x) is a nonlinear function.

Now we obtain new iterative schemes by using the decomposition techniques of [11]. The main idea of this decomposition technique is applied to find the solution in the following series form.

(8) $$x=\sum_{i=0}^{\infty }x_{i.}$$

The nonlinear operator N(x) can be decomposed as shown in the following equation

(9) $$N\left(x\right)=N\left(x_{0}\right)+\sum_{i=1}^{\infty }\left\{N\left(\sum_{j=0}^{i}x_{j}\right)-N\left(\sum_{j=0}^{i-1}x_{j}\right)\right\}.$$

From Eq. (6), (8), (9), we get,

(10) $$\left\{ \begin{array}{c} x_{0}=c\\ x_{1}=N\left(x_{0}\right)\\ x_{2}=N\left(x_{1}\right)-N\left(x_{0}\right)\\ \vdots \\ x_{m}=N\left(\sum_{j=0}^{m}x_{j}\right)-N\left(\sum_{j=0}^{m-1}x_{j}\right) \end{array}\right.$$

From Eq. (10), we have

(11) $$X_{m}=x_{1}+x_{2}\cdots +x_{m+1}=N\left(x_{0}+x_{1}+x_{2}+\cdots +x_{m}\right).$$

Where

(12) $$\lim_{m\to \infty }X_{m}=x.$$

Now for m=0

(13) $$x\approx X_{m}=x_{0}=c=\gamma .$$

For m=1

(14) $$x\approx X_{m}=x_{0}+x_{1}=\;\gamma +N\left(x_{0}\right).$$

So, from Eq. (8) and (11)

(15) $$x_{1}=\;N\left(x_{0}\right)=-\frac{4\left(h\left(x_{0}\right)+f\left(\gamma \right)p\left(\gamma \right)\right)}{4p^{\prime }\left(\gamma \right)f\left(\gamma \right)+\left\{f^{\prime }\left(\gamma \right)+2f^{\prime }\left(\frac{\gamma +x_{0}}{2}\right)+f^{\prime }\left(x_{0}\right)\right\}p\left(\gamma \right)}.$$

From Eq. (4) and by using the idea of suggested by Yun [27].

(16) $$h\left(x_{0}\right)=0.$$

Substituting Eq. (16) in (15) and combining with Eq. (14) and (11).

(18) $$x=x_{0}+x_{1}=\;\gamma -\frac{4f\left(\gamma \right)p\left(\gamma \right)}{4p^{\prime }\left(\gamma \right)f\left(\gamma \right)+\left\{f^{\prime }\left(\gamma \right)+2f^{\prime }\left(\gamma \right)+f^{\prime }\left(\gamma \right)\right\}p\left(\gamma \right)},$$

Which yields to the

(19) $$x=\;\gamma -\frac{f\left(\gamma \right)p\left(\gamma \right)}{p^{\prime }\left(\gamma \right)f\left(\gamma \right)+f^{\prime }\left(\gamma \right)p\left(\gamma \right)}.$$

Eq. (19) allows us to establish the following one-step iterative scheme for solving the nonlinear equation as:

Algorithm 2.1 For a given $x_{0}$, compute the approximate solution $x_{n+1}$ by the following iterative scheme.

$$x_{n+1}=\;x_{n}-\frac{f\left(x_{n}\right)p\left(x_{n}\right)}{p^{\prime }\left(x_{n}\right)f\left(x_{n}\right)+f^{\prime }\left(x_{n}\right)p\left(x_{n}\right)}$$

From Eq. (19), we have

(20) $$x_{0}+x_{1}-\;\gamma =\;-\frac{f\left(\gamma \right)p\left(\gamma \right)}{p^{\prime }\left(\gamma \right)f\left(\gamma \right)+f^{\prime }\left(\gamma \right)p\left(\gamma \right)}.$$

Now using (4) and (7) with the help of the idea given by using Yun [33], we have

(21) $$h\left(x_{0}+x_{1}\right)=f\left(x_{0}+x_{1}\right)p\left(\gamma \right)-f\left(\gamma \right)p\left(\gamma \right)-\left(x_{0}+x_{1}-\gamma \right)\left[p^{\prime }\left(\gamma \right)f\left(\gamma \right)+\left\{\frac{f^{\prime }\left(\gamma \right)+2f^{\prime }\left(\frac{\gamma +x_{0}+x_{1}}{2}\right)+f^{\prime }\left(x_{0}+x_{1}\right)}{4}\right\}p\left(\gamma \right)\right]=$$

(22) $$f\left(x_{0}+x_{1}\right)p\left(\gamma \right)-f\left(\gamma \right)p\left(\gamma \right)+\left(\frac{f\left(\gamma \right)p\left(\gamma \right)}{p^{\prime }\left(\gamma \right)f\left(\gamma \right)+f^{\prime }\left(\gamma \right)p\left(\gamma \right)}\right)\left[p^{\prime }\left(\gamma \right)f\left(\gamma \right)+\left\{\frac{f^{\prime }\left(\gamma \right)+2f^{\prime }\left(\frac{\gamma +x_{0}+x_{1}}{2}\right)+f^{\prime }\left(x_{0}+x_{1}\right)}{4}\right\}p\left(\gamma \right)\right].$$

From Eq. (8) and (23) we have

(23) $$x_{1}+x_{2}=N\left(x_{0}+x_{1}\right)=-\frac{4\left(h\left(x\right)+f\left(\gamma \right)p\left(\gamma \right)\right)}{4p^{\prime }\left(\gamma \right)f\left(\gamma \right)+\left\{f^{\prime }\left(\gamma \right)+2f^{\prime }\left(\frac{\gamma +x}{2}\right)+f^{\prime }\left(x\right)\right\}p\left(\gamma \right)}=-\frac{f\left(\gamma \right)p\left(\gamma \right)}{p^{\prime }\left(\gamma \right)f\left(\gamma \right)+f^{\prime }\left(\gamma \right)p\left(\gamma \right)}-\;\frac{4f\left(x_{0}+x_{1}\right)p\left(\gamma \right)}{p^{\prime }\left(\gamma \right)f\left(\gamma \right)+\left(f^{\prime }\left(\gamma \right)+2f^{\prime }\left(\frac{\gamma +x_{0}+x_{1}}{2}\right)+f^{\prime }\left(x_{0}+x_{1}\right)\right)p\left(\gamma \right)}.$$

For m=2

(24) $$x\approx X_{2}=x_{0}+x_{1}+x_{2}=\;\gamma +N\left(x_{0}+x_{1}\right).$$

Now by combining Eq. (7), (23) and (24) we get,

(25) $$x=\;\gamma -\frac{f\left(\gamma \right)p\left(\gamma \right)}{p^{\prime }\left(\gamma \right)f\left(\gamma \right)+f^{\prime }\left(\gamma \right)p\left(\gamma \right)}-\frac{4f\left(x_{0}+x_{1}\right)p\left(\gamma \right)}{4p^{\prime }\left(\gamma \right)f\left(\gamma \right)+\left(f^{\prime }\left(\gamma \right)+2f^{\prime }\left(\frac{\gamma +x_{0}+x_{1}}{2}\right)+f^{\prime }\left(x_{0}+x_{1}\right)\right)p\left(\gamma \right)}.$$

Using the above relation, we conclude to suggest the following two-step iterative method for solving nonlinear Eq. (1).

Algorithm 2.2 For a given $x_{0}$, compute the approximate solution $x_{n+1}$ by the following iterative scheme:

$$y_{n}=x_{n}-\frac{f\left(x_{n}\right)p\left(x_{n}\right)}{p^{\prime }\left(x_{n}\right)f\left(x_{n}\right)+f^{\prime }\left(x_{n}\right)p\left(x_{n}\right)},$$

$$x_{n+1}=y_{n}-\frac{4f\left(y_{n}\right)p\left(x_{n}\right)}{4p^{\prime }\left(x_{n}\right)f\left(x_{n}\right)+\left(f^{\prime }\left(x_{n}\right)+2f^{\prime }\left(\frac{x_{n}+y_{n}}{2}\right)+f^{\prime }\left(y_{n}\right)\right)p\left(x_{n}\right)}.$$

From Eq. (25), we have

(26) $$x_{0}+x_{1}+x_{2}-\;\gamma =\;-\frac{f\left(\gamma \right)p\left(\gamma \right)}{p^{\prime }\left(\gamma \right)f\left(\gamma \right)+f^{\prime }\left(\gamma \right)p\left(\gamma \right)}-\frac{4f\left(x_{0}+x_{1}\right)p\left(\gamma \right)}{4p^{\prime }\left(\gamma \right)f\left(\gamma \right)+\left(f^{\prime }\left(\gamma \right)+2f^{\prime }\left(\frac{\gamma +x_{0}+x_{1}}{2}\right)+f^{\prime }\left(x_{0}+x_{1}\right)\right)p\left(\gamma \right)}.$$

Now from Eq. (4) and using the design of Yun [33], we have

(27) $$h\left(x_{0}+x_{1}+x_{2}\right)=f\left(x_{0}+x_{1}+x_{2}\right)p\left(\gamma \right)-f\left(\gamma \right)p\left(\gamma \right)-\left(x_{0}+x_{1}+x_{2}-\gamma \right)\times \left[p^{\prime }\left(\gamma \right)f\left(\gamma \right)+\left\{\frac{f^{\prime }\left(\gamma \right)+2f^{\prime }\left(\frac{\gamma +x_{0}+x_{1}+x_{2}}{2}\right)+f^{\prime }\left(x_{0}+x_{1}+x_{2}\right)}{4}\right\}p\left(\gamma \right)\right]$$

(28) $$=f\left(x_{0}+x_{1}+x_{2}\right)p\left(\gamma \right)-f\left(\gamma \right)p\left(\gamma \right)-\left(-\frac{f\left(\gamma \right)p\left(\gamma \right)}{p^{\prime }\left(\gamma \right)f\left(\gamma \right)+f^{\prime }\left(\gamma \right)p\left(\gamma \right)}-\frac{4f\left(x_{0}+x_{1}\right)p\left(\gamma \right)}{4p^{\prime }\left(\gamma \right)f\left(\gamma \right)+\left(f^{\prime }\left(\gamma \right)+2f^{\prime }\left(\frac{\gamma +x_{0}+x_{1}}{2}\right)+f^{\prime }\left(x_{0}+x_{1}\right)\right)p\left(\gamma \right)}\right)\times \left[p^{\prime }\left(\gamma \right)f\left(\gamma \right)+\left\{\frac{f^{\prime }\left(\gamma \right)+2f^{\prime }\left(\frac{\gamma +x_{0}+x_{1}+x_{2}}{2}\right)+f^{\prime }\left(x_{0}+x_{1}+x_{2}\right)}{4}\right\}p\left(\gamma \right)\right],$$

and

(29) $$x_{1}+x_{2}=N\left(x_{0}+x_{1}\right)=-\frac{f\left(\gamma \right)p\left(\gamma \right)}{p^{\prime }\left(\gamma \right)f\left(\gamma \right)+f^{\prime }\left(\gamma \right)p\left(\gamma \right)}-\;\frac{4f\left(x_{0}+x_{1}\right)p\left(\gamma \right)}{4p^{\prime }\left(\gamma \right)f\left(\gamma \right)+\left(f^{\prime }\left(\gamma \right)+2f^{\prime }\left(\frac{\gamma +x_{0}+x_{1}}{2}\right)+f^{\prime }\left(x_{0}+x_{1}\right)\right)p\left(\gamma \right)}-\frac{4f\left(x_{0}+x_{1}\right)p\left(\gamma \right)}{4p^{\prime }\left(\gamma \right)f\left(\gamma \right)+\left(f^{\prime }\left(\gamma \right)+2f^{\prime }\left(\frac{\gamma +x_{0}+x_{1}+x_{2}}{2}\right)+f^{\prime }\left(x_{0}+x_{1}+x_{2}\right)\right)p\left(\gamma \right)}.$$

For m=3

(30) $$x\approx X_{2}=x_{0}+x_{1}+x_{2}+x_{3}=\;\gamma +N\left(x_{0}+x_{1}+x_{3}\right).$$

So by combining Eqs. (7), (26) and (33) we get the following

(31) $$x=\;\gamma -\frac{f\left(\gamma \right)p\left(\gamma \right)}{g^{\prime }\left(\gamma \right)f\left(\gamma \right)+f^{\prime }\left(\gamma \right)g\left(\gamma \right)}-\frac{4f\left(x_{0}+x_{1}\right)p\left(\gamma \right)}{4p^{\prime }\left(\gamma \right)f\left(\gamma \right)+\left(f^{\prime }\left(\gamma \right)+2f^{\prime }\left(\frac{\gamma +x_{0}+x_{1}}{2}\right)+f^{\prime }\left(x_{0}+x_{1}\right)\right)p\left(\gamma \right)}-\frac{4f\left(x_{0}+x_{1}\right)p\left(\gamma \right)}{4p^{\prime }\left(\gamma \right)f\left(\gamma \right)+\left(f^{\prime }\left(\gamma \right)+2f^{\prime }\left(\frac{\gamma +x_{0}+x_{1}+x_{2}}{2}\right)+f^{\prime }\left(x_{0}+x_{1}+x_{2}\right)\right)p\left(\gamma \right)}.$$

Above relation allows us to suggest the following three-step iterative method for solving Eq. (1).

Algorithm 2.3 For a given $x_{0}$, calculate the approximate solution $x_{n+1}$ by the following iterative schemes:

$$\begin{array}{c} y_{n}=x_{n}-\frac{f\left(x_{n}\right)p\left(x_{n}\right)}{p^{\prime }\left(x_{n}\right)f\left(x_{n}\right)+f^{\prime }\left(x_{n}\right)p\left(x_{n}\right)},\\ z_{n}=y_{n}-\frac{4f\left(y_{n}\right)p\left(x_{n}\right)}{4p^{\prime }\left(x_{n}\right)f\left(x_{n}\right)+\left(f^{\prime }\left(x_{n}\right)+2f^{\prime }\left(\frac{x_{n}+y_{n}}{2}\right)+f^{\prime }\left(y_{n}\right)\right)p\left(x_{n}\right)},\\ x_{n+1}=z_{n}-\frac{4f\left(z_{n}\right)p\left(x_{n}\right)}{4p^{\prime }\left(x_{n}\right)f\left(x_{n}\right)+\left(f^{\prime }\left(x_{n}\right)+2f^{\prime }\left(\frac{x_{n}+z_{n}}{2}\right)+f^{\prime }\left(z_{n}\right)\right)p\left(x_{n}\right)} \end{array}$$

From Algorithms 2.1, 2.2 and 2.3, we suggest some iterative method for solving nonlinear Eq. (1) by using different values of auxiliary function. By choosing suitable auxiliary functions diversify the main recurrence relation to obtain the best solution for nonlinear Eq. (1).

We consider the two auxiliary functions which are $p\left(x\right)=e^{-\omega x}$ and$\;p\left(x\right)=e^{-\omega \frac{f(x)}{f^{\prime }\left(x\right)}}$**.** When we choose$\;p\left(x\right)=e^{-\omega x}$**,** and $p\left(x\right)=e^{-\omega \frac{f(x)}{f^{\prime }\left(x\right)}},$ these will provide us with Algorithms from 2.4 to 2.6 and Algorithms 2.7 to 2.9 respectively. Some of these methods are well known and treated as special cases of our main recurrence relations.

Algorithm 2.4 For a given $x_{0}$, compute the approximate solution $x_{n+1}$ by the following iterative scheme:

$$x_{n+1}=x_{n}-\frac{f\left(x_{n}\right)}{f^{\prime }\left(x_{n}\right)-\omega f\left(x_{n}\right)}$$

Well known Newton method [19] is a special case of Algorithm 2.4, when we take ω=0.

Algorithm 2.5 For a given $x_{0}$, compute the approximate solution $x_{n+1}$ by the following iterative schemes:

$$\begin{array}{c} y_{n}=x_{n}-\frac{f\left(x_{n}\right)}{f^{\prime }\left(x_{n}\right)-\omega f\left(x_{n}\right)},\\ x_{n+1}=y_{n}-\frac{4f\left(y_{n}\right)}{f^{\prime }\left(x_{n}\right)+2f^{\prime }\left(\frac{x_{n}+y_{n}}{2}\right)+f^{\prime }\left(y_{n}\right)-4\omega f\left(x_{n}\right)}. \end{array}$$

Algorithm 2.6 For a given $x_{0}$, compute the approximate solution $x_{n+1}$ by the following iterative schemes:

$$\begin{array}{c} y_{n}=x_{n}-\frac{f\left(x_{n}\right)}{f^{\prime }\left(x_{n}\right)-\omega f\left(x_{n}\right)},\\ z_{n}=y_{n}-\frac{4f\left(y_{n}\right)}{f^{\prime }\left(x_{n}\right)+2f^{\prime }\left(\frac{x_{n}+y_{n}}{2}\right)+f^{\prime }\left(y_{n}\right)-4\omega f\left(x_{n}\right)},\\ x_{n+1}=z_{n}-\frac{4f\left(z_{n}\right)}{f^{\prime }\left(x_{n}\right)+2f^{\prime }\left(\frac{x_{n}+z_{n}}{2}\right)+f^{\prime }\left(z_{n}\right)-4\omega f\left(x_{n}\right)}. \end{array}$$

Algorithm 2.7 For a given $x_{0}$, compute the approximate solution $x_{n+1}$ by the following iterative scheme:

$$x_{n+1}=x_{n}-\frac{f\left(x_{n}\right){\left(f^{\prime }\left(x_{n}\right)\right)}^{2}}{{\left(f^{\prime }\left(x_{n}\right)\right)}^{3}-\omega \left((f^{\prime }{\left(x_{n}\right)}^{2}+f\left(x_{n}\right)f^{\prime \prime }\left(x_{n}\right)\right)f\left(x_{n}\right)}$$

Well known Newton method is a special case of Algorithm 2.7. when we take ω=0.

Algorithm 2.8 For a given $x_{0}$, compute the approximate solution $x_{n+1}$ by the following iterative schemes:

$$\begin{array}{c} y_{n}=x_{n}-\frac{f\left(x_{n}\right){\left(f^{\prime }\left(x_{n}\right)\right)}^{2}}{{\left(f^{\prime }\left(x_{n}\right)\right)}^{3}-\omega \left({\left(f^{\prime }\left(x_{n}\right)\right)}^{2}+f\left(x_{n}\right)f^{\prime \prime }\left(x_{n}\right)\right)f\left(x_{n}\right)},\\ x_{n+1}=y_{n}-\frac{4f\left(y_{n}\right){\left(f^{\prime }\left(x_{n}\right)\right)}^{2}}{\left(f^{\prime }\left(x_{n}\right)+2f^{\prime }\left(\frac{x_{n}+y_{n}}{2}\right)+f^{\prime }\left(y_{n}\right)\right){\left(f^{\prime }\left(x_{n}\right)\right)}^{2}-4\omega \left((f^{\prime }{\left(x_{n}\right)}^{2}+f\left(x_{n}\right)f^{\prime \prime }\left(x_{n}\right)\right)f\left(x_{n}\right)}. \end{array}$$

Algorithm 2.9 For a given $x_{0}$, compute the approximate solution $x_{n+1}$ by the following iterative schemes:

$$\begin{array}{c} y_{n}=x_{n}-\frac{f\left(x_{n}\right){\left(f^{\prime }\left(x_{n}\right)\right)}^{2}}{{\left(f^{\prime }\left(x_{n}\right)\right)}^{3}-\omega \left((f^{\prime }{\left(x_{n}\right)}^{2}+f\left(x_{n}\right)f^{\prime \prime }\left(x_{n}\right)\right)f\left(x_{n}\right)},\\ z_{n}=y_{n}-\frac{4f\left(y_{n}\right){\left(f^{\prime }\left(x_{n}\right)\right)}^{2}}{\left(f^{\prime }\left(x_{n}\right)+2f^{\prime }\left(\frac{x_{n}+y_{n}}{2}\right)+f^{\prime }\left(y_{n}\right)\right)(f^{\prime }{\left(x_{n}\right)}^{2}-4\omega \left({\left(f^{\prime }\left(x_{n}\right)\right)}^{2}+f\left(x_{n}\right)f^{\prime \prime }\left(x_{n}\right)\right)f\left(x_{n}\right)},\\ x_{n+1}=z_{n}-\frac{4f\left(z_{n}\right){\left(f^{\prime }\left(x_{n}\right)\right)}^{2}}{\left(f^{\prime }\left(x_{n}\right)+2f^{\prime }\left(\frac{x_{n}+z_{n}}{2}\right)+f^{\prime }\left(z_{n}\right)\right)(f^{\prime }{\left(x_{n}\right)}^{2}-4\omega \left((f^{\prime }{\left(x_{n}\right)}^{2}+f\left(x_{n}\right)f^{\prime \prime }\left(x_{n}\right)\right)f\left(x_{n}\right)}. \end{array}$$

**Remark:** By varying the value of ω, we can generate distinct classes of iterative schemes from these newly developed methods. Optimal results can be achieved by selecting the value of that maximizes the denominator of the corrector function.

**Table:** Nomenclature.

**Symbols**	**Descriptions**
IT	Number of Iterations
$\;x_{0}$	Initial Guess
$x_{n}$	Current iterate in the iterative method
$x_{n+1}$	Next iterate (updated value)
ε	Tolerance for convergence
$\;|x_{n+1}-x_{n}|$	Absolute Error
COC	Computational order of convergence
TOC	Time of Computations
NM	Newton method
Alg	Algorithm
DIV	Divergence

### Convergence analysis

This section is devoted to analyzing the convergence behavior of the proposed iterative methods, presented earlier as Algorithm (2.3), by utilizing Taylor series approach.

Theorem 3.1 *Let* α∈I *be a simple zero of sufficiently differentiable function* f:I⊆R→R *for an open interval* I. *If* $x_{0\;}$*is sufficiently close to α, then iterative method define in Algorithm 2.3 has at least fourth-order convergence*.

**Proof.** Let α be a simple zero of f(x). Then by expanding $f(x_{n})$ and $f^{\prime }\left(x_{n}\right)$, in Taylor’s series about α we have

(32) $$f\left(x_{n}\right)=f^{\prime }\left(\alpha \right)\left[e_{n}+c_{2}e_{n}^{2}+c_{3}e_{n}^{3}+c_{4}e_{n}^{4}+c_{5}e_{n}^{5}+c_{6}e_{n}^{6}+O\left(e_{n}^{7}\right)\right],$$

and

(33) $$f^{\prime }\left(x_{n}\right)=f^{\prime }\left(\alpha \right)\left[1+2c_{2}e_{n}+3c_{3}e_{n}^{2}+4c_{4}e_{n}^{3}+5c_{5}e_{n}^{4}+6c_{6}e_{n}^{5}+O\left(e_{n}^{6}\right)\right].$$

Where

$$c_{k}=\frac{1}{k!}\frac{f^{\left(k\right)}}{f^{\prime }\left(\alpha \right)},k=2,3,\ldots ande_{n}=x-a$$

Now we expand $\left(x_{n}\right)p\left(x_{n}\right),\;f{}^{\prime }\left(x_{n}\right)p\left(x_{n}\right)$ and $f\left(x_{n}\right)p{}^{\prime }\left(x_{n}\right)$ by Taylor series, we obtain,

(34) $$f\left(x_{n}\right)p\left(x_{n}\right)=f^{\prime }\left(\alpha \right)\left[p\left(\alpha \right)e_{n}+\left(p\left(\alpha \right)+c_{2}p^{\prime }\left(\alpha \right)\right)e_{n}^{2}+p_{1}e_{n}^{3}+O\left(e_{n}^{4}\right)\right],$$

(35) $$f\left(x_{n}\right)p^{\prime }\left(x_{n}\right)=f^{\prime }\left(\alpha \right)\left[p^{\prime }\left(\alpha \right)e_{n}+\left(p^{\prime }\left(\alpha \right)+c_{2}p^{\prime \prime }\left(\alpha \right)\right)e_{n}^{2}+p_{2}e_{n}^{3}+O\left(e_{n}^{4}\right)\right],$$

and

(36) $$f{\left(x_{n}\right)}^{\prime }p\left(x_{n}\right)=f^{\prime }\left(\alpha \right)\left[p\left(\alpha \right)+\left(p^{\prime }\left(\alpha \right)+2c_{2}p\left(\alpha \right)\right)e_{n}+p_{1}e_{n}^{2}+p_{3}e_{n}^{3}+O\left(e_{n}^{4}\right)\right].$$

Where

(37) $$p_{1}=\frac{p^{\prime \prime }\left(\alpha \right)}{2}+c_{3}p\left(\alpha \right)+c_{2}p{}^{\prime }\left(\alpha \right),$$

(38) $$p_{2}=\frac{p^{\prime \prime \prime }\left(\alpha \right)}{2}+c_{3}p^{\prime }\left(\alpha \right)+c_{2}p{}^{\prime \prime }\left(\alpha \right),$$

and

(39) $$p_{3}=\frac{p^{\prime \prime \prime }\left(\alpha \right)}{6}+4c_{4}p\left(\alpha \right)+c_{2}gp^{{}^{\prime \prime }}\left(\alpha \right)+3c_{3}p^{\prime }\left(\alpha \right).$$

From Eq. (34), (35), (36), we get

(40) $$\frac{f\left(x_{n}\right)p\left(x_{n}\right)}{f^{\prime }\left(x_{n}\right)p\left(x_{n}\right)+f\left(x_{n}\right)p^{\prime }\left(x_{n}\right)}=e_{n}-\left(\frac{p^{\prime }\left(\alpha \right)}{p\left(\alpha \right)}+c_{2}\right)e_{n}^{2}-\left(\frac{p^{\prime \prime }\left(\alpha \right)}{p\left(\alpha \right)}c_{2}^{2}+2c_{3}-2\frac{p^{\prime }\left(\alpha \right)}{p\left(\alpha \right)}c_{2}+2{\left(\frac{p^{\prime }\left(\alpha \right)}{p\left(\alpha \right)}\right)}^{2}+2c_{2}^{2}\right)e_{n}^{3}+O\left(e_{n}^{4}\right).$$

Using Eq. (40), we have

(41) $$y_{n}=\alpha +e_{n}+\left(\frac{p^{\prime }\left(\alpha \right)}{p\left(\alpha \right)}+c_{2}\right)e_{n}^{2}+\left(\frac{p^{\prime \prime }\left(\alpha \right)}{p\left(\alpha \right)}+2c_{3}-2\frac{p^{\prime }\left(\alpha \right)}{p\left(\alpha \right)}c_{2}-2{\left(\frac{p^{\prime }\left(\alpha \right)}{p\left(\alpha \right)}\right)}^{2}-2c_{2}^{2}\right)e_{n}^{3}+O\left(e_{n}^{4}\right).$$

Now Expanding $f(y_{n})$ by Taylor’s series about α and using Eq. (41), we have

(42) $$f\left(y_{n}\right)=f^{\prime }\left(\alpha \right)\left[\left(\frac{p^{\prime }\left(\alpha \right)}{p\left(\alpha \right)}+c_{2}\right)e_{n}^{2}+\left(\frac{p^{\prime \prime }\left(\alpha \right)}{p\left(\alpha \right)}+2c_{3}-2\frac{p^{\prime }\left(\alpha \right)}{p\left(\alpha \right)}c_{2}-2{\left(\frac{p^{\prime }\left(\alpha \right)}{p\left(\alpha \right)}\right)}^{2}-2c_{2}^{2}\right)e_{n}^{3}+O\left(e_{n}^{4}\right)\right].$$

Similarly, we get

(43) $$f^{\prime }\left(y_{n}\right)=f^{\prime }\left(\alpha \right)\left[1+2c_{2}\left(\frac{p^{\prime }\left(\alpha \right)}{p\left(\alpha \right)}+c_{2}\right)e_{n}^{2}+2c_{2}\left(\frac{p^{\prime \prime }\left(\alpha \right)}{p\left(\alpha \right)}+2c_{3}-2\frac{p^{\prime }\left(\alpha \right)}{p\left(\alpha \right)}c_{2}-2{\left(\frac{p^{\prime }\left(\alpha \right)}{p\left(\alpha \right)}\right)}^{2}-2c_{2}^{2}\right)e_{n}^{3}+O\left(e_{n}^{4}\right)\right].$$

Now we find $f\left(y_{n}\right)\;p\left(x_{n}\right)$ by expanding with Taylor’s series

(44) $$f\left(y_{n}\right)\;p\left(x_{n}\right)=f^{\prime }\left(\alpha \right)\left[\left(c_{2}p\left(\alpha \right)+p{}^{\prime }\left(\alpha \right)\right)e_{n}^{2}-\left(\frac{{\left(p{}^{\prime }\left(\alpha \right)\right)}^{2}}{p\left(\alpha \right)}+\frac{p^{\prime }\left(\alpha \right)}{p\left(\alpha \right)}c_{2}-p^{\prime \prime }\left(\alpha \right)-2c_{3}p\left(\alpha \right)+c_{2}^{2}p\left(\alpha \right)\right)e_{n}^{3}+O\left(e_{n}^{4}\right)\right].$$

By Taylor’s series, we obtain the expansion of $f(\frac{x_{n}+y_{n}}{2})$ as,

(45) $$f\left(\frac{x_{n}+y_{n}}{2}\right)\;=f^{\prime }\left(\alpha \right)\left[1+c_{2}e_{n}+\frac{1}{2}\left(4c_{2}\frac{p^{\prime }\left(\alpha \right)}{p\left(\alpha \right)}+4c_{2}^{2}+3c_{3}\right)e_{n}^{2}+O\left(e_{n}^{3}\right)\right].$$

Using Eq. (33), (43), (45), we get

(46) $$\begin{array}{c} \left[f^{\prime }\left(x_{n}\right)+2f\left(\frac{x_{n}+y_{n}}{2}\right)+f^{\prime }\left(y_{n}\right)\right]p\left(x_{n}\right)=f^{\prime }\left(a\right)\\ \left[4p\left(a\right)+\left(4p^{\prime }\left(a\right)+5c_{2}p\left(a\right)\right)e_{n}+\frac{1}{4}\left(8p^{\prime \prime }\left(a\right)+21c_{3}p\left(a\right)+32c_{2}p^{\prime }\left(a\right)+12c_{2}^{2}p\left(a\right)\right)e_{n}^{2}+O\left(e_{n}^{3}\right)\right] \end{array}$$

Using Eqs. (35), (45) and (46), we get

(47) $$\begin{array}{c} \frac{4f\left(y_{n}\right)p\left(x_{n}\right)}{\left[f^{\prime }\left(x_{n}\right)+2f\left(\frac{x_{n}+y_{n}}{2}\right)+f^{\prime }\left(y_{n}\right)\right]p\left(x_{n}\right)+4f\left(x_{n}\right)p^{\prime }\left(x_{n}\right)}\left(\frac{p^{\prime \prime }\left(a\right)}{p\left(a\right)}+c_{2}\right)e_{n}^{2}-\frac{1}{4}\\ \left[12{\left(\frac{p^{\prime \prime }\left(a\right)}{p\left(a\right)}\right)}^{2}+17c_{2}\frac{p^{\prime }\left(a\right)}{p\left(a\right)}-4\frac{p^{\prime \prime }\left(a\right)}{p\left(a\right)}-8c_{3}+13c_{2}^{2}\right]e_{n}^{3}+O\left(e_{n}^{4}\right) \end{array}$$

Using Eqs. (41) and (47), we get

(48) $$z=\alpha +\left({\left(\frac{p^{\prime \prime }\left(\alpha \right)}{p\left(\alpha \right)}\right)}^{2}+2c_{2}\frac{p^{\prime }\left(\alpha \right)}{p\left(\alpha \right)}+c_{2}^{2}\right)e_{n}^{3}+\frac{1}{8}\left(16\frac{p^{\prime \prime }\left(\alpha \right)}{p\left(\alpha \right)}c_{2}+25\frac{p^{\prime }\left(\alpha \right)}{p\left(\alpha \right)}-56\frac{{\left(p^{\prime }\left(\alpha \right)\right)}^{2}}{p{\left(\alpha \right)}^{3}}c_{2}+16\frac{p^{\prime }\left(\alpha \right)p^{\prime \prime }\left(\alpha \right)}{p{\left(\alpha \right)}^{3}}-48c_{2}^{2}\frac{p^{\prime }\left(\alpha \right)}{p\left(\alpha \right)}+25c_{2}c_{3}-32{\left(\frac{p^{\prime \prime }\left(\alpha \right)}{p\left(\alpha \right)}\right)}^{3}-24c_{2}^{3}\right)e_{n}^{4}+O\left(e_{n}^{5}\right).$$

Now using Eq. (48), we have

(49) $$f\left(z\right)=f^{\prime }\left(\alpha \right)\left[\left({\left(\frac{p^{\prime \prime }\left(\alpha \right)}{p\left(\alpha \right)}\right)}^{2}+2c_{2}\frac{p^{\prime }\left(\alpha \right)}{p\left(\alpha \right)}+c_{2}^{2}\right)e_{n}^{3}+\frac{1}{8}\left(16\frac{{\left(p^{\prime \prime }\left(\alpha \right)\right)}^{3}}{p\left(\alpha \right)}c_{2}+25\frac{p^{\prime }\left(\alpha \right)}{p\left(\alpha \right)}c_{3}+16\frac{p^{\prime }\left(\alpha \right)p^{\prime \prime }\left(\alpha \right)}{p{\left(\alpha \right)}^{3}}-56\frac{{\left(p^{\prime }\left(\alpha \right)\right)}^{2}}{p{\left(\alpha \right)}^{3}}c_{2}-48c_{2}^{2}\frac{p^{\prime }\left(\alpha \right)}{p\left(\alpha \right)}+25c_{2}c_{3}-32{\left(\frac{p^{\prime \prime }\left(\alpha \right)}{p\left(\alpha \right)}\right)}^{3}-24c_{2}^{3}\right)e_{n}^{4}+O\left(e_{n}^{5}\right)\right],$$

and

(50) $$f^{\prime }\left(z\right)=f^{\prime }\left(\alpha \right)\left[1+\left(2c_{2}{\left(\frac{p^{\prime \prime }\left(\alpha \right)}{p\left(\alpha \right)}\right)}^{2}+2c_{2}\frac{p^{\prime }\left(\alpha \right)}{p\left(\alpha \right)}+c_{2}^{2}\right)e_{n}^{3}+\frac{1}{4}c_{2}\left(16\frac{{\left(p^{\prime \prime }\left(\alpha \right)\right)}^{3}}{p\left(\alpha \right)}c_{2}+25\frac{p^{\prime }\left(\alpha \right)}{p\left(\alpha \right)}c_{3}+16\frac{p^{\prime }\left(\alpha \right)p^{\prime \prime }\left(\alpha \right)}{p{\left(\alpha \right)}^{3}}-56\frac{{\left(p^{\prime }\left(\alpha \right)\right)}^{2}}{p{\left(\alpha \right)}^{3}}c_{2}-48c_{2}^{2}\frac{p^{\prime }\left(\alpha \right)}{p\left(\alpha \right)}+25c_{2}c_{3}-32{\left(\frac{p^{\prime \prime }\left(\alpha \right)}{p\left(\alpha \right)}\right)}^{3}+24c_{2}^{3}\right)e_{n}^{4}+O\left(e_{n}^{5}\right)\right].$$

Again using Eq. (48), (49) and (50), we get

(51) $$\begin{array}{c} \left[f^{\prime }\left(x_{n}\right)+2f\left(\frac{x_{n}+z_{n}}{2}\right)+f^{\prime }\left(z_{n}\right)\right]p\left(x_{n}\right)=f^{\prime }\left(a\right)\\ \left[4p\left(\alpha \right)+4\left(p^{\prime }\left(a\right)+c_{2}p\left(a\right)\right)e_{n}+\left(2p^{\prime \prime }\left(a\right)+4c_{2}p^{\prime }\left(a\right)+\frac{9}{2}c_{3}p\left(a\right)\right)e_{n}^{2}+O\left(e_{n}^{3}\right)\right] \end{array}$$

Using Eq. (49) and (51), we obtain

(52) $$\begin{array}{c} \frac{4f\left(z_{n}\right)p\left(x_{n}\right)}{\left[f^{\prime }\left(x_{n}\right)+2f\left(\frac{x_{n}+z_{n}}{2}\right)+f^{\prime }\left(z_{n}\right)\right]p\left(x_{n}\right)+4f\left(x_{n}\right)p^{\prime }\left(x\right)}=\left({\left(\frac{p^{\prime }\left(a\right)}{p\left(a\right)}\right)}^{2}+2c_{2}\frac{p^{\prime }\left(a\right)}{p\left(a\right)}+c_{2}^{2}\right)e_{n}^{3}-\frac{1}{8}\\ \left(40{\left(\frac{p^{\prime \prime }\left(a\right)}{p\left(a\right)}\right)}^{3}+80{\left(\frac{p^{\prime \prime }\left(a\right)}{p\left(a\right)}\right)}^{3}c_{2}+72c_{2}^{2}\frac{p^{\prime }\left(a\right)}{p\left(a\right)}-\frac{{\left(p^{\prime }\left(a\right)\right)}^{2}}{p{\left(a\right)}^{2}}c_{2}-25\frac{p^{\prime }\left(a\right)}{p\left(a\right)}-16\frac{p^{\prime }\left(a\right)p^{\prime \prime }\left(a\right)}{p{\left(a\right)}^{3}}-25c_{2}c_{3}+32c_{2}^{3}\right)e_{n}^{4}+O\left(e_{n}^{5}\right) \end{array}$$

By using Eq. (47) and (52), we get the result as

(53) $$x_{n+1}=\alpha +\left(3{\left(\frac{p^{\prime }\left(\alpha \right)}{p\left(\alpha \right)}\right)}^{2}c_{2}+3c_{2}^{2}\frac{p^{\prime }\left(\alpha \right)}{p\left(\alpha \right)}+{\left(\frac{p^{\prime }\left(\alpha \right)}{p\left(\alpha \right)}\right)}^{3}+c_{2}^{3}\right)e_{n}^{4}+O\left(e_{n}^{5}\right).$$

Finally, we get the error equation from Eq. (53)

(54) $$e_{n+1}=\left(3{\left(\frac{p^{\prime }\left(\alpha \right)}{p\left(\alpha \right)}\right)}^{2}c_{2}+3c_{2}^{2}\frac{p^{\prime }\left(\alpha \right)}{p\left(\alpha \right)}+{\left(\frac{p^{\prime }\left(\alpha \right)}{p\left(\alpha \right)}\right)}^{3}+c_{2}^{3}\right)e_{n}^{4}+O\left(e_{n}^{5}\right).$$

Error equation (56) shows that the scheme mentioned in the Algorithm 2.3 generates at least fourth order convergent iterative methods for the nonlinear equations.

### Method validation

In this section, we implement numerical simulations for some real-life applications to supply significant validation of the proposed iterative schemes expressed as Algorithms 2.4 - 2.9. Comparison is made with existing methods with the classical Newton method (NM), Chun’s methods, CH1 [8], CH2 [8], CH3 [9], CH4 [9] and SH [21]. The error tolerance is set to $\varepsilon =10^{-13}$, while the precision is set to 400. The numerical results are calculated and analyzed in this way additionally. In each Table, we collect the number of iterations required for any technique applied to meet the mentioned error tolerance, the computational order of convergence, the absolute error, the function's absolute value at the final step of the proceedure. These numerical explorations were performed on a machine with Windows 11 (128 bit) with 24.0 GB of RAM, and an Intel Core i7–1065G7 CPU (1.50 GHz) processor. Stopping criteria for computer programming is shown as:

$$\left|x_{n+1}-x_{n}\right|<\varepsilon .$$

The computational order of convergence (COC) approximated as

$$coc=\frac{ln\left(\left|x_{n+1}-x_{n}\right|/\left|x_{n}-x_{n-1}\right|\right)}{ln\left(\left|x_{n}-x_{n-1}\right|/\left|x_{n-1}-x_{n-2}\right|\right)}$$

**Fig. 1:**
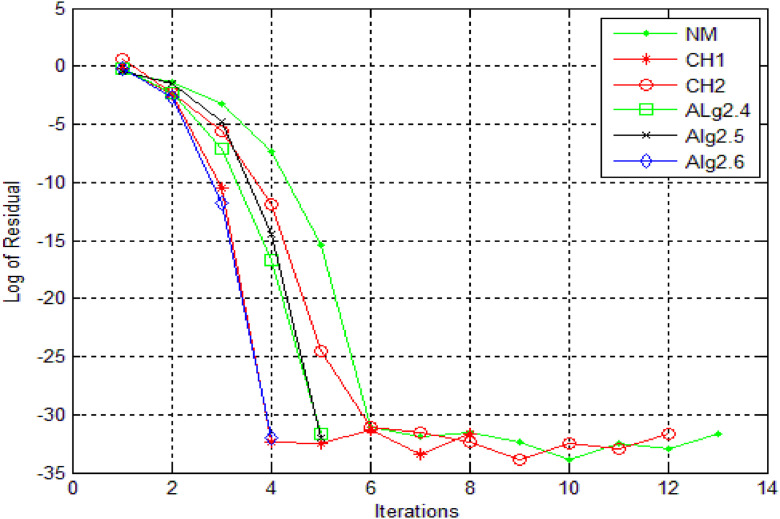
Log of residue of Example 1.

**Fig. 2:**
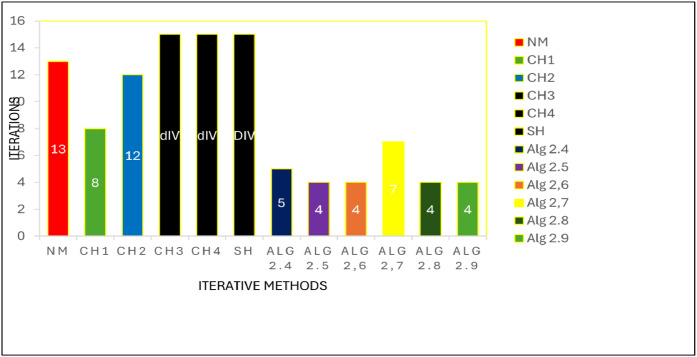
Comparison of iterations for Example 1.

**Fig. 3:**
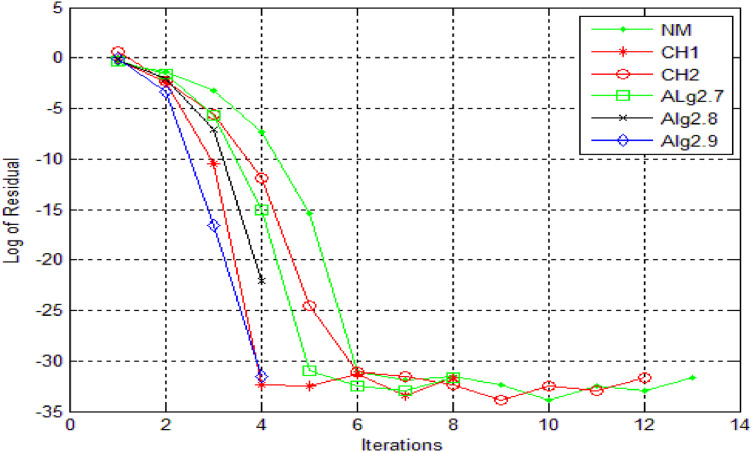
Log of residue of Example 1.

**Fig. 4:**
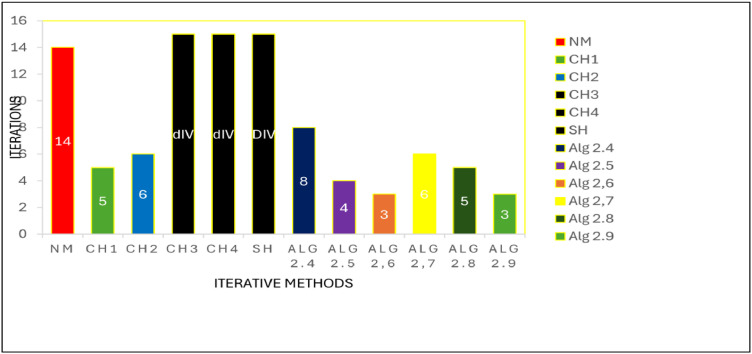
Comparison of iterations for Example 2.

Numerical results, for examples 1 and 2 are shown in TABLE 1, Table 2 while log of residues are shown by graph in Figs. 1,2,Fig. 3, Fig. 4 respectively by using Algorithms 2.4–2.9. IT stands for number of iterations, $|x_{n+1}-x_{n}|$ is the difference of the last two consecutive iterations, COC is also given in Tables and in the last column TOC is expressed for CPU time taking one second as unit.

**TABLE 1:** **(**Numerical result for Example 4.1).

Method	ω	$x_{n+1}$	IT	$|x_{n+1}-x_{n}|$	COC	TOC
NM	-	0.100997929685740	13	1.8001e-14	1.98361039461551	0.0160
CH1	-	0.100997929685740	8	1.8001e-14	3.10445656730423	0.0150
CH2	-	0.100997929685740	12	1.8001e-14	3.00232300230455	0.0160
CH3	-	DIV	-	-	-	-
CH4	-	DIV	-	-	-	-
SH	-	DIV	-	-	-	-
Alg2.4	0.4	0.100997929685740	5	1.8000e-14	2.34553635649529	0.0140
Alg2.5	0.4	0.100997929685740	4	2.4686e-10	2.96798171496893	0.0140
Alg 2.6	−0.2	0.100997929685740	4	1.3000e-14	3.96124106685813	0.0140
Alg2.7	0.54	0.100997929685740	7	1.8000e-14	3.80904621045617	0.0150
Alg2.8	−0.2	0.100997929685740	4	2.4686e-10	2.97015872405991	0.0140
Alg 2.9	0.1	0.100997929685740	4	2.1000e-14	4.03557680849725	0.0140

**Table 2:** **(**Numerical result for example 4.2).

Method	ω	$x_{n+1}$	IT	$|x_{n+1}-x_{n}|$	COC	TOC
NM	-	−0.317061774531111	14	3.5000000000e-14	2.07199263041623	0.0160
CH1	-	−0.317061774531066	5	3.0000000000e-14	3.11394152433442	0.0150
CH2	-	−0.317061774531127	6	9.5000000000e-14	2.98394752854687	0.0160
CH3	-	DIV	-	-	-	-
CH4	-	DIV	-	-	-	-
SH	-	DIV	-	-	-	-
Alg2.4	−0.6	−0.317061774531037	8	1.6159053000e-08	2.00212508311349	0.0140
Alg2.5	−0.2	−0.317061774531073	4	1.5090000000e-11	2.98394501593417	0.0140
Alg 2.6	−0.3	−0.317061774531111	3	2.5169423986e-05	3.90774890438257	0.0140
Alg2.7	−0.6	−0.317061774531066	6	3.7127510000e-09	2.00137483225598	0.0150
Alg2.8	−1	−0.317061774531073	5	5.7914419000e-08	2.98474714409496	0.0140
Alg 2.9	−0.2	−0.317061774531142	3	1.4224339000e-08	3.99711813534491	0.0140

DIV stands for divergence.

Example 1 Analyze short-term population dynamics by assuming the growth of population grows continuously with time at a rate of proportional to the number present at that time. Let N(t) denotes the number at time t and λ denotes the constant birth rate proportion. Supposing a uniform immigration ratev, then the population satisfies the differential equation

$$\frac{dN\left(t\right)}{dt}=\lambda N\left(t\right)+v.$$

The solution is given by:

$$N\left(t\right)=N_{0}e^{\lambda t}+\frac{v}{\lambda }\left(e^{\lambda t}-1\right).$$

Suppose a certain population consists on N(0)=1,000,000 residents initially, that 435,000 residents relocate into the community in the first year and that N(1)=1,564,000 residents are present at the end of one year. To settle this population, we have to gain λ from the following equation

$$1,564,000=1,000,000\;e^{\lambda }+\frac{435,000}{\lambda }\left(e^{\lambda }-1\right).$$

We use $x_{0}=1,$ as a priori estimate and approximated above problem up to 15 decimal digits is 0.100997929685750.

A comparative analysis of the residual fall for each method in Example 1 is presented in Fig. 1. This visualization enables a detailed evaluation of the methods' performance, revealing that the newly developed methods demonstrate a substantially faster rate of residual fall. This accelerated convergence underscores the improved efficacy and precision of the new methods, making them an asset for solving related problems.

A comprehensive comparison of various iterative methods is presented in Fig. 2, showcasing their performance on Example 1. Notably, all methods were implemented with identical stopping criteria and initial guesses, ensuring a fair evaluation. The results unequivocally demonstrate that the newly derived iterative methods significantly outperform existing methods, achieving faster convergence and greater accuracy. Moreover, it is evident that the CH3, CH4, and SH methods fail to converge for this specific problem, diverging and failing to attain the required accuracy for the root. In contrast, the newly developed methods exhibit robust performance, consistently approaching the root with precision.

Residual fall related to all methods, for example 1 can also be viewed in Fig. 3. One can compare the methods and can obtain the result that newly derived methods have faster fall.

Example 2 Assume that a particle has zero initial momentum on smooth inclined plane whose angle θ is changing at a constant rate

$$\frac{d\theta }{dt}=\omega <0,$$

At the end of *t* seconds, the position of the object is given by

$$x\left(t\right)=-\frac{g}{2\omega^{2}}\left(\frac{e^{\omega t}-e^{-\omega t}}{2}-sin\;\omega t\right).$$

Imagine the particle has traversed a distance 1.7ft in1sec. We have to find the rate at which angle θ changes. Assuming that$\;g=32.17ft/sec^{2}$. The solution of problem approximated to the 15 decimals digits is −0.317061774531088. We us $x_{0}=3,$ as initial guess for this example.

A comparative analysis of iterative methods is presented in Fig. 4, where Example 2 is solved using identical stopping criteria and initial guesses for each method. The results illustrate the superior performance of newly derived iterative methods, which demonstrate accelerated convergence and enhanced accuracy compared to existing methods. Furthermore, the CH3, CH4, and SH methods exhibit divergence for this problem, failing to achieve the prescribed accuracy for the root. Conversely, the novel methods display consistent and robust convergence, accurately approximating the root.

**Fig. 5:**
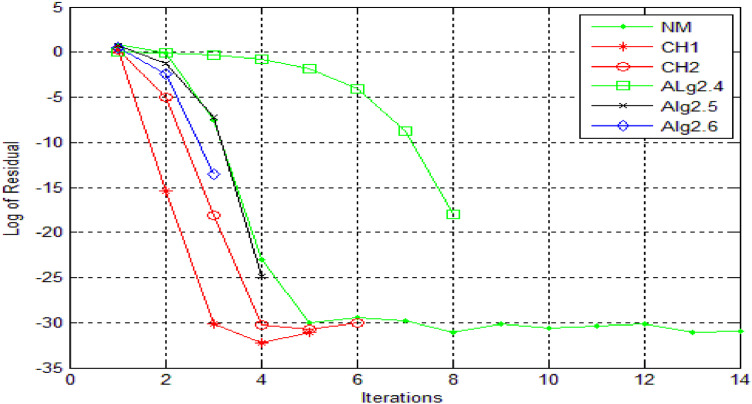
log of residue of Example 2.

The residual fall associated with each method for Example 2 is illustrated in Fig. 5, allowing for a comprehensive comparison of the methods. Upon examination, it is evident that the newly derived methods exhibit a significantly faster rate of residual fall compared to existing methods. This indicates that the new approaches converge more rapidly to the solution, showcasing their enhanced efficiency and accuracy.

**Fig. 6:**
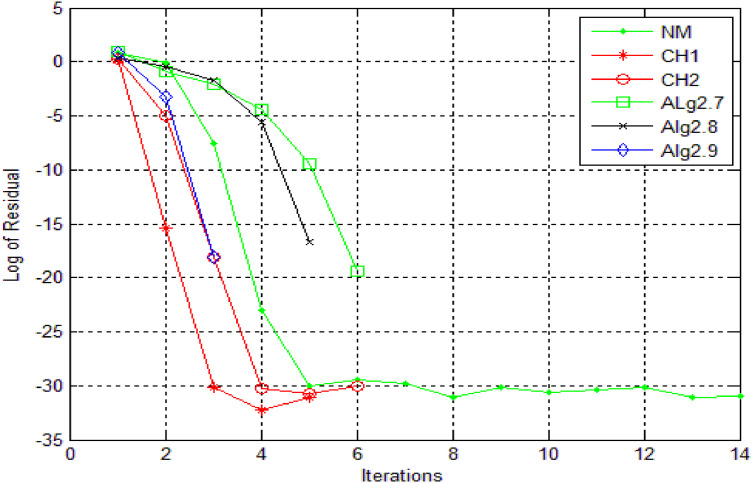
Log of residue of Example 2.

Residual fall related to all methods, for example 2 can be viewed in Fig. 6. One can compare the methods and can obtain the result that newly derived methods have faster fall. The choice of numerical method for solving nonlinear equations plays a crucial role in achieving accurate and efficient solutions. While single-step methods have their strengths, multi-step methods offer distinct advantages in handling highly nonlinear equations, including improved convergence, increased accuracy and robustness.

Efficiency index is the combination of order of convergence and number of function evaluations per iteration which is $E.I=p^{1/m},$ where p is order of convergence of the method while m in the number of functional evaluations per iteration [32]. A higher order of convergence combined with fewer function evaluations leads to an improved efficiency index and a more optimal iterative method. However, researchers must sometimes address serious limitations of existing methods, as such methods may fail to obtain solutions for certain classes of nonlinear problems. Therefore, the efficiency index may be compromised. As in this article draw backs of Newton method and Newton type methods are overcome. The result can be observed in Numerical calculations in Tables which indicate that some methods diverge. Given methods in this article are converging to an exact solution while some methods such as CH3 CH4 and SH are diverging. Here is the detail in Table 3 regarding efficiency index of newly derived methods.

**Table 3:** (Efficiency index of suggested methods).

Methods	Order of Convergence	Functional Evaluations	Efficiency Index
Algorithm 2.4	2	2	1.4142
Algorithm 2.5	3	5	1.2457
Algorithm 2.6	4	8	1.1892
Algorithm 2.7	2	3	1.2600
Algorithm 2.8	3	6	1.2009
Algorithm 2.9	4	9	1.1606

### Fractal analysis of the proposed algorithms

Fractal geometry offers an insightful way to visualize the dynamics of iterative methods for solving nonlinear equations. When applied to complex-valued initial guesses, iterative schemes reveal the basins of attraction corresponding to different roots of the nonlinear equation. The boundary regions between basins often display complex fractal structures. In this section, we explore the fractal patterns generated using Algorithms 2.4 to 2.9 on a range of nonlinear functions.

All basin-of-attraction fractals presented in this work were generated using MATLAB (R2021a) for various test polynomials. The complex plane was discretized over the square domain Re(z),Im(z)∈[−2,2], using a uniform 300 × 300 grid of initial points. For each initial value$z_{0}$, the corresponding iterative scheme was applied with a relaxation parameter ω=0.5 and a maximum of 50 iterations. At each iteration, the stopping criterion was based on the residual condition∣ $|f(z_{k})|<10^{-6}$, while iterations were terminated prematurely if the denominator of the update formula satisfied ∣ $|\text{denominator}|<10^{-12}$, to avoid numerical instability. After termination, each initial point was assigned to the root with minimum distance among the known exact roots. Points satisfying $|f(z)|<10^{-4}$were classified as convergent; otherwise, they were treated as non-convergent. Distinct hues were assigned to different roots, and the color intensity was scaled according to the number of iterations required to reach convergence, with brighter shades indicating faster convergence. Non-convergent points were displayed in a uniform dark tone. Circular contours were additionally drawn around the true roots to highlight local convergence behavior. The same domain, grid resolution, stopping criteria, and color-mapping strategy were used across all algorithms and test functions to ensure a fair and reproducible comparison.

The following nonlinear equations were selected to analyze and compare the fractal convergence behavior of the proposed algorithms:

Example 3: $f_{1}\left(z\right)=z^{2}-1.$
Example 4**:** $f_{2}\left(z\right)=z^{3}-1.$
Example 5**:** $f_{3}\left(z\right)=z^{4}-1.$

These test functions were selected to represent polynomial equations of increasing degree which allow the proposed methods to be examined in the presence of multiple real and complex roots. It also assess their convergence behaviour and basin structures as the algebraic complexity of the problem increases.

**Fig. 7:**
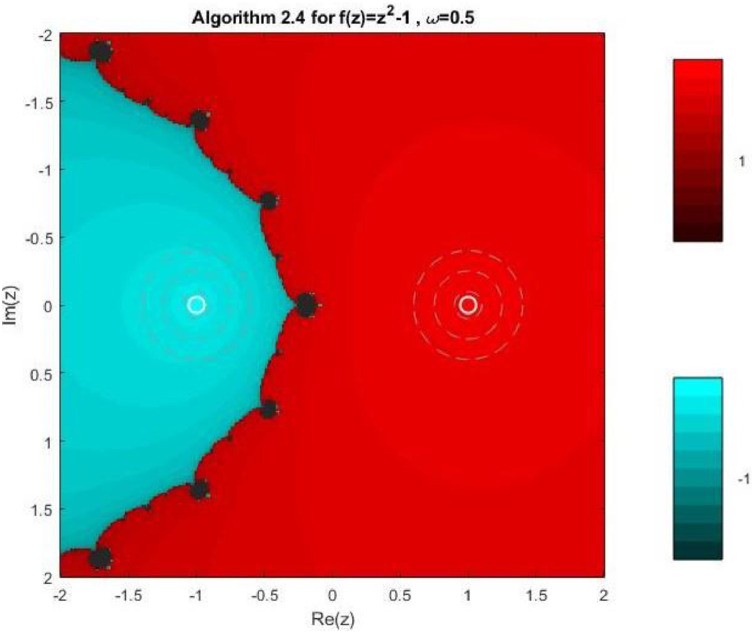
Fractal for Algorithm 2.4.

**Fig. 8:**
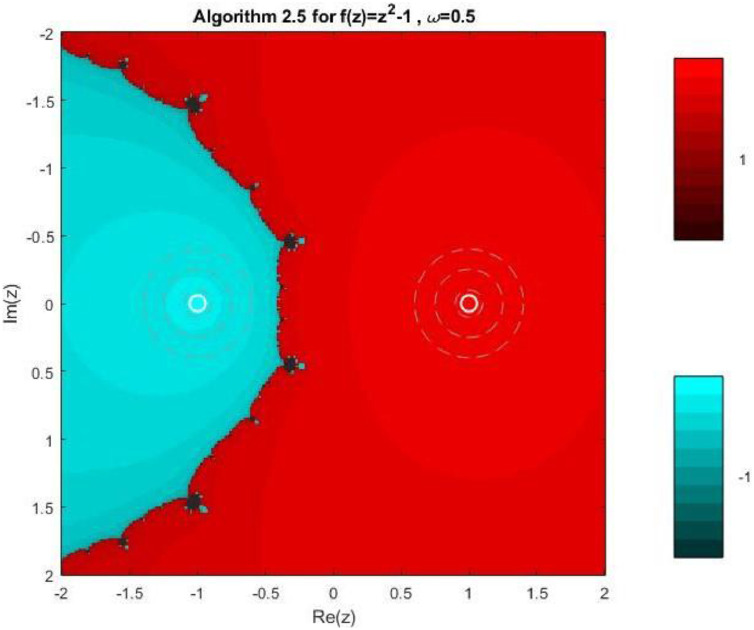
Fractal for Algorithm 2.5.

**Fig. 9:**
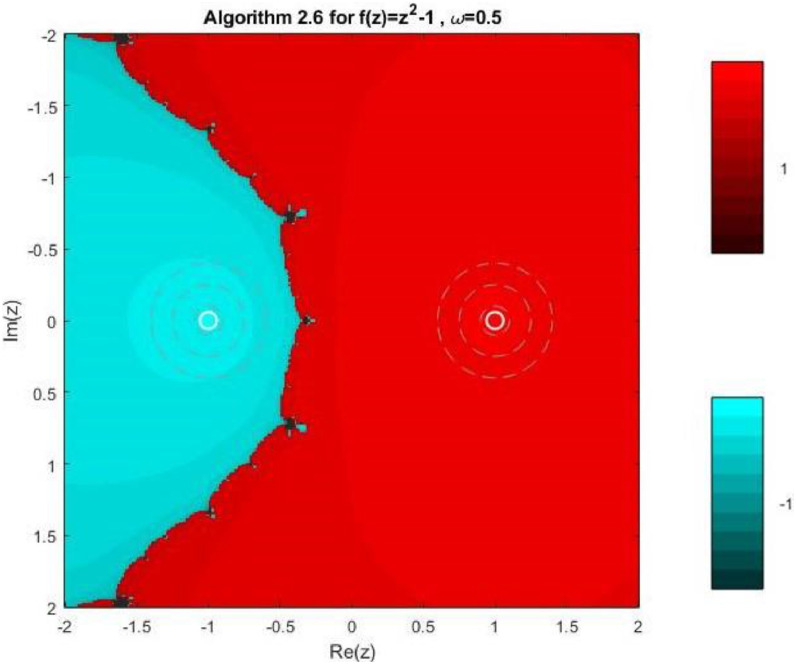
Fractal for Algorithm 2.6.

**Fig. 10:**
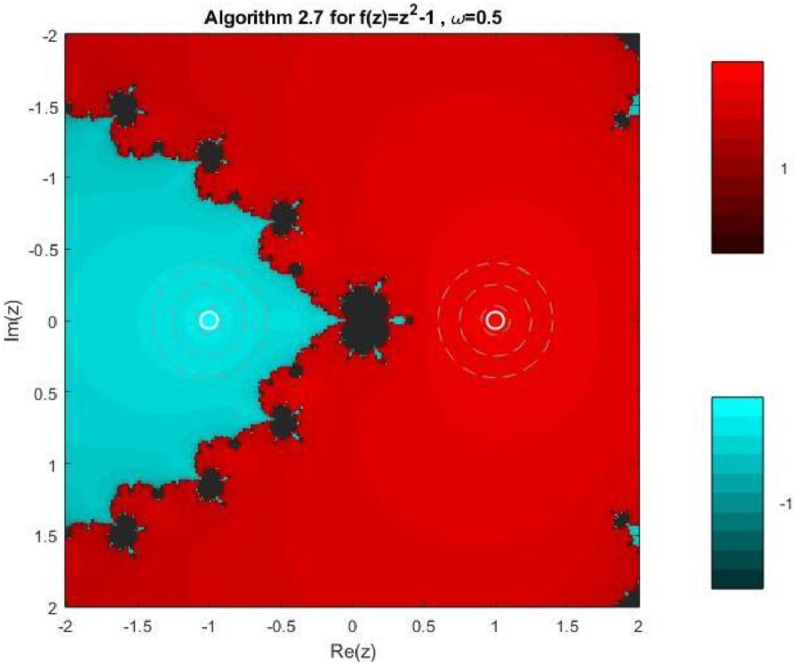
Fractal for Algorithm 2.7.

**Fig. 11:**
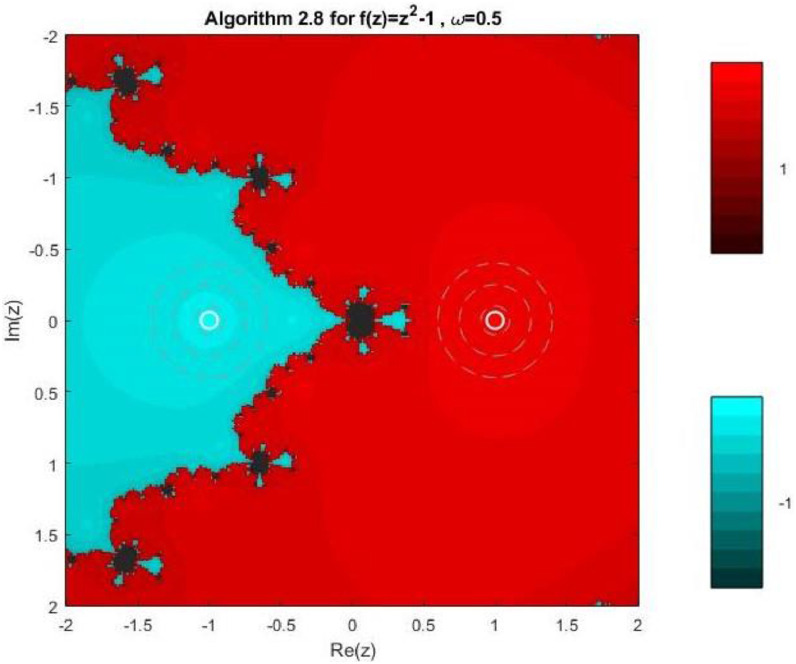
Fractal for Algorithm 2.8.

**Fig. 12:**
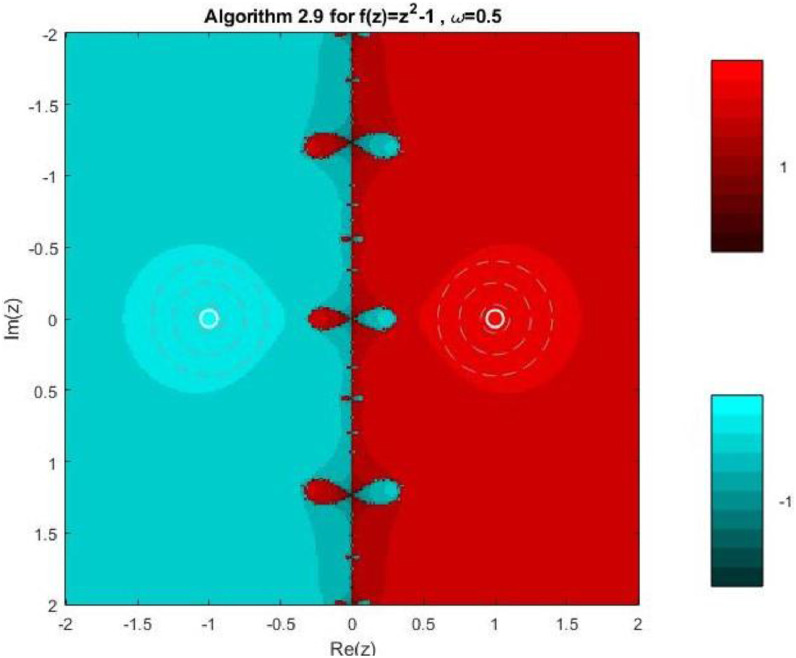
Fractal for Algorithm 2.9.

Example 3 To investigate the convergence behaviour of the proposed iterative methods, we have generated fractal plots for the Quadratic equation $f_{1}\left(z\right)=z^{2}-1.$ using Algorithms 2.4 to 2.9 presented in Fig. 7, Fig. 8, Fig. 9, Fig. 10, Fig. 11, Fig. 12. These fractals illustrate the basins of attraction corresponding to two simple roots z=+1,−1in the complex plane. The colour scheme represents which root a given initial point converges to, while the brightness reflects the speed of convergence. These visualizations offer a clear and intuitive comparison of the convergence efficiency, stability, and sensitivity of the algorithms. As shown in Fig. 7, Algorithm 2.4 produces two well-defined basins separated by a curved fractal boundary. The convergence is relatively fast near the true roots, as indicated by the lighter colour intensity aroundz=1,−1.. However, the boundary between the basins exhibits noticeable irregularity, suggesting sensitivity to initial guesses in this region. Darker pixels concentrated along the boundary indicate slower convergence and higher iteration counts. Fig. 8 demonstrates that Algorithm 2.5 slightly improves the basin geometry compared to Algorithm 2.4. The basins become smoother near the roots, and the region of slow convergence is reduced. This behaviour reflects the enhanced corrective step that is incorporated in the algorithm. It stabilizes convergence for a wider set of initial points while preserving the overall basin structure. For the next algorithm 2.6, Fig. 9, further refines the basin boundaries. The attraction regions corresponding to each root expand, and the fractal boundary becomes less irregular. The colour intensity distribution suggests a reduction in the average number of iterations required for convergence, indicating improved efficiency over Algorithms 2.4 and 2.5.

Fig. 10 illustrates that Algorithm 2.7 introduces more pronounced structural changes near the basin boundary. Although the main basins remain dominant, small localized regions of slow convergence appear around the separating curve. These features reflect the influence of higher-order derivative information, which enhances convergence near the roots but may increase sensitivity along critical boundary regions. The fractal representation in last two plots of this group, reveals a significant improvement in convergence behaviour. In particular, Algorithm 2.9 exhibits a nearly vertical and sharply defined boundary separating the two basins. The basins themselves are more uniform in colour, indicating faster and more consistent convergence across the complex plane. The presence of small symmetric structures near the imaginary axis reflects the high-order corrective steps, but these do not compromise overall stability. Overall, the fractal analysis clearly demonstrates a progressive enhancement in convergence performance from Algorithm 2.4 to Algorithm 2.9. While all methods successfully converge to the correct roots, the higher-order algorithms, especially Algorithms 2.8 and 2.9, exhibit:

• Smoother and more regular basin boundaries,
• Reduced regions of slow convergence,
• Greater robustness with respect to the choice of initial guesses.

These observations confirm that the proposed multi-step and higher-order schemes provide superior convergence characteristics even for simple nonlinear problems, thereby justifying their application to more complex equations.

**Fig. 13:**
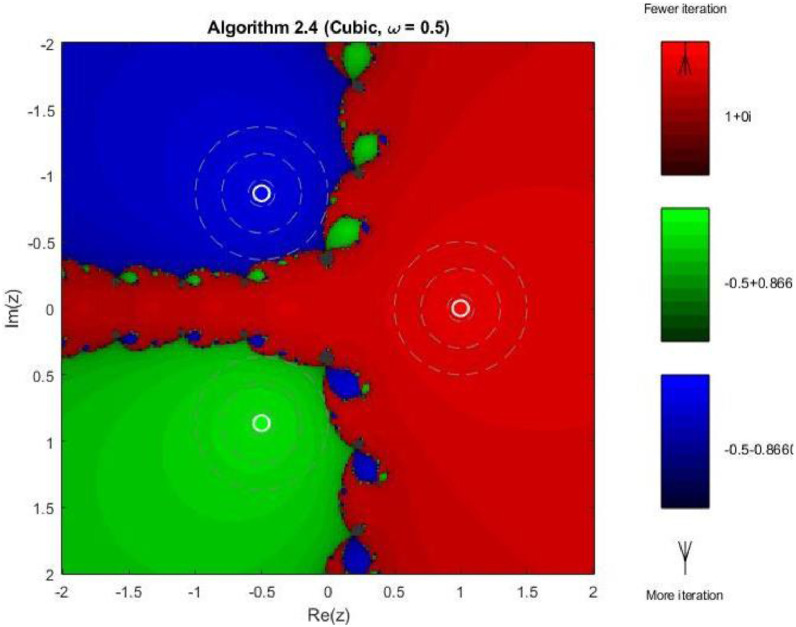
Fractal for Algorithm 2.4.

**Fig. 14:**
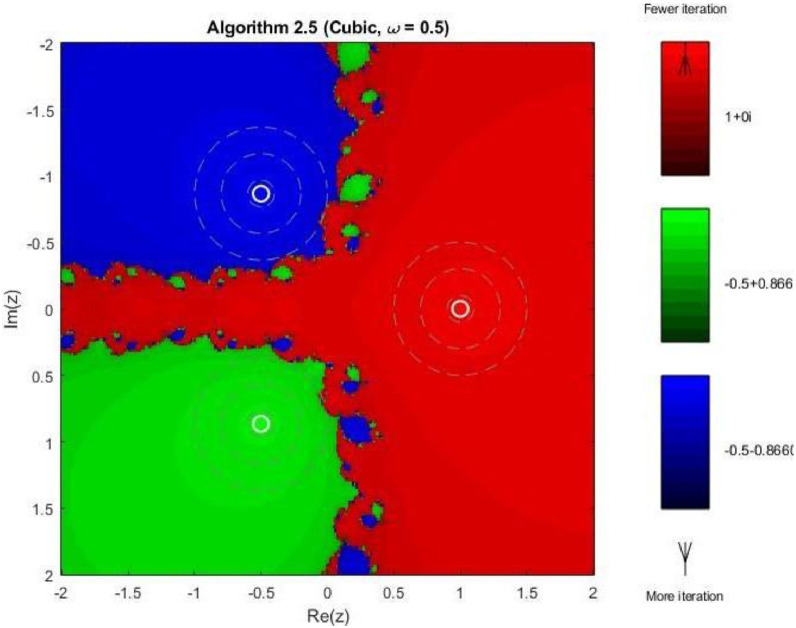
Fractal for Algorithm 2.5.

**Fig. 15:**
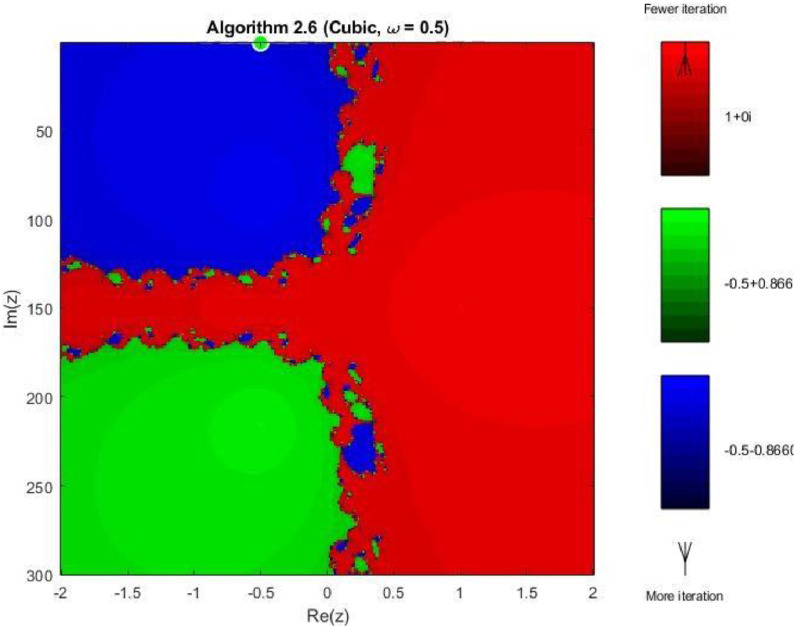
Fractal for Algorithm 2.6.

**Fig. 16:**
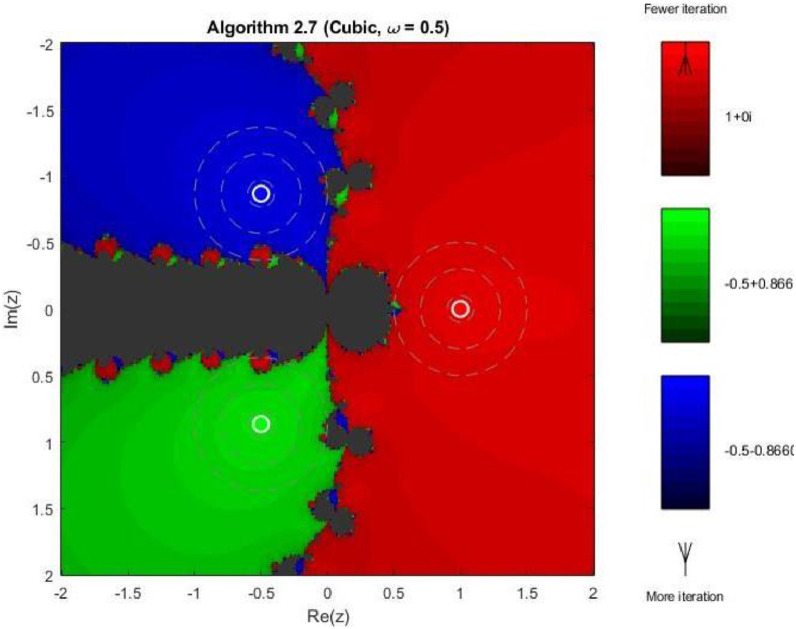
Fractal for Algorithm 2.7.

**Fig. 17:**
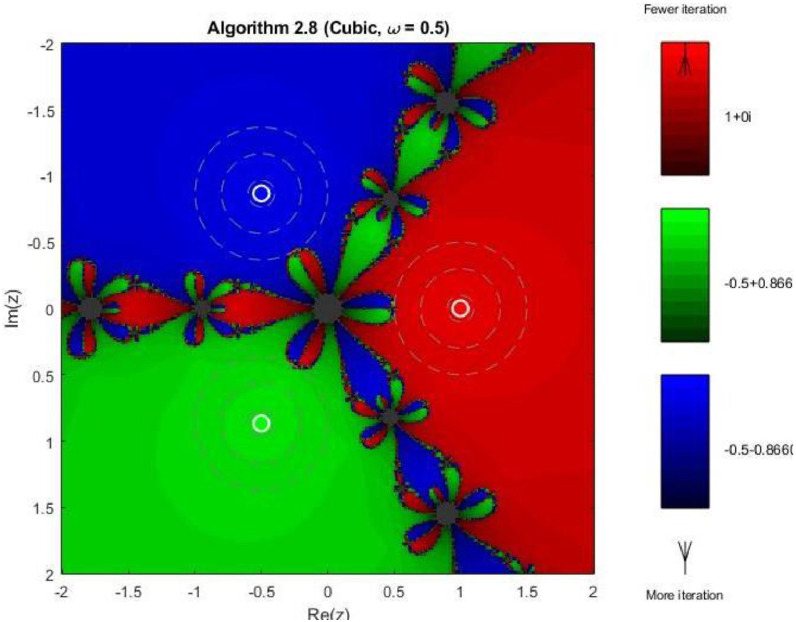
Fractal for Algorithm 2.8.

**Fig. 18:**
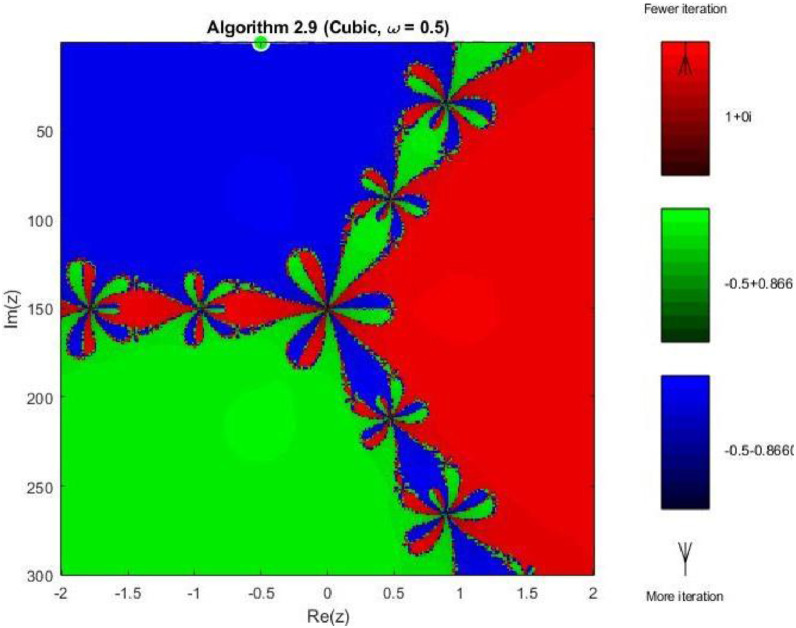
Fractal for Algorithm 2.9.

Example 4 To further examine the convergence behaviour of the proposed iterative schemes, fractal plots are generated for the cubic polynomial $f_{2}\left(z\right)=z^{3}-1$ using Algorithms 2.4 to 2.9, as illustrated in Fig. 13, Fig. 14, Fig. 15, Fig. 16, Fig. 17, Fig. 18. This equation has three distinct roots given by, $z_{1}=1,\;\;\;\;\;z_{2}=-\frac{1}{2}+\frac{\sqrt{3}}{2}\iota ,\;\;\;\;z_{2}=-\frac{1}{2}-\frac{\sqrt{3}}{2}\iota$. Consequently, three main basins of attraction appear in the complex plane, each represented by a distinct colour. The brightness of each colour reflects the number of iterations required for convergence, where lighter shades correspond to faster convergence. These fractal patterns provide clear insight into the stability, convergence speed, and sensitivity of the proposed algorithms with respect to initial guesses.

As observed in Fig. 13, Algorithm 2.4 produces three clearly distinguishable basins of attraction corresponding to the three roots of the cubic polynomial. Although convergence near the roots is relatively fast, the basin boundaries are highly irregular and exhibit pronounced fractal structures. These complex boundaries indicate strong sensitivity to initial guesses, particularly in regions equidistant from multiple roots. Fig. 14 shows that Algorithm 2.5 slightly improves the basin geometry. The attraction regions become more uniform, and the regions of slow convergence along the basin boundaries are reduced. Nevertheless, noticeable fractal irregularities persist, especially near the intersection points of the three basins, reflecting moderate instability for certain initial values.

In 3rd figure of this group, demonstrates further refinement of the basin structures. The basins expand more symmetrically, and the fractal boundary regions become thinner. The color intensity suggests a decrease in the average number of iterations compared to Algorithms 2.4 and 2.5, indicating enhanced convergence efficiency and stability. The next Fig. 16 reveals a distinctive change in convergence behavior for Algorithm 2.7. While the three main basins remain dominant, larger dark regions appear along the central boundary, indicating slower convergence or delayed stabilization for some initial guesses. This behavior can be attributed to the increased complexity of the iterative correction, which enhances convergence near roots but introduces sensitivity in transition regions.

As shown in Fig. 17, Algorithm 2.8 produces highly structured and symmetric basin boundaries with petal-like patterns near the separatrix. Despite the increased geometric complexity, the basins themselves are well defined, and convergence within each basin is generally rapid. The refined boundary patterns reflect the higher-order nature of the algorithm and its strong dependence on initial conditions near critical points. In Fig. 18 illustrates that Algorithm 2.9 achieves the most uniform and stable convergence behavior among all tested methods. The basins of attraction are larger and smoother, with sharply defined boundaries and fewer regions of slow convergence. The color intensity remains relatively uniform within each basin, confirming faster and more consistent convergence across the complex plane. From a comprehensive perspective the fractal analysis for the cubic polynomial demonstrates a clear progression in convergence performance from Algorithm 2.4 to Algorithm 2.9. While lower-order methods exhibit highly irregular basin boundaries and greater sensitivity to initial guesses, the higher-order schemes

**Fig. 19:**
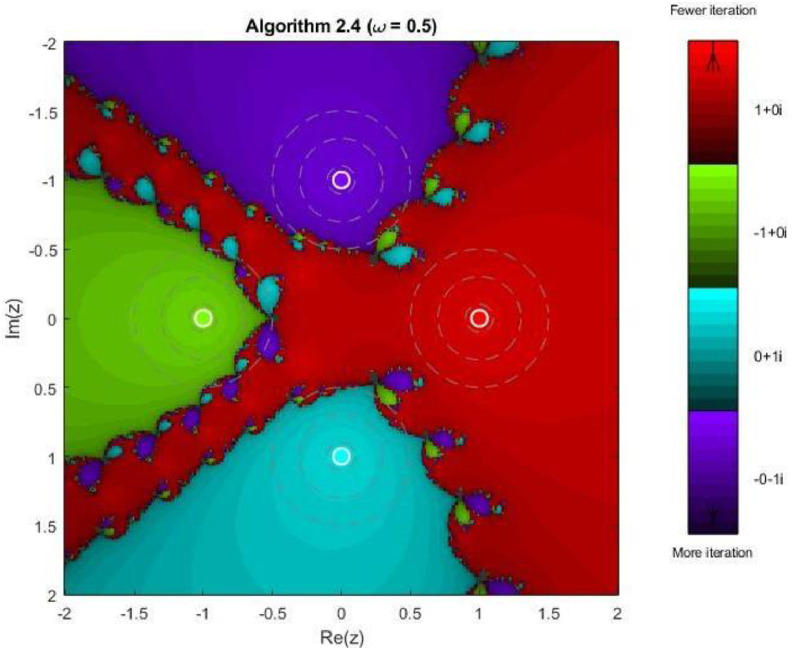
Fractal for Algorithm 2.4.

**Fig. 20:**
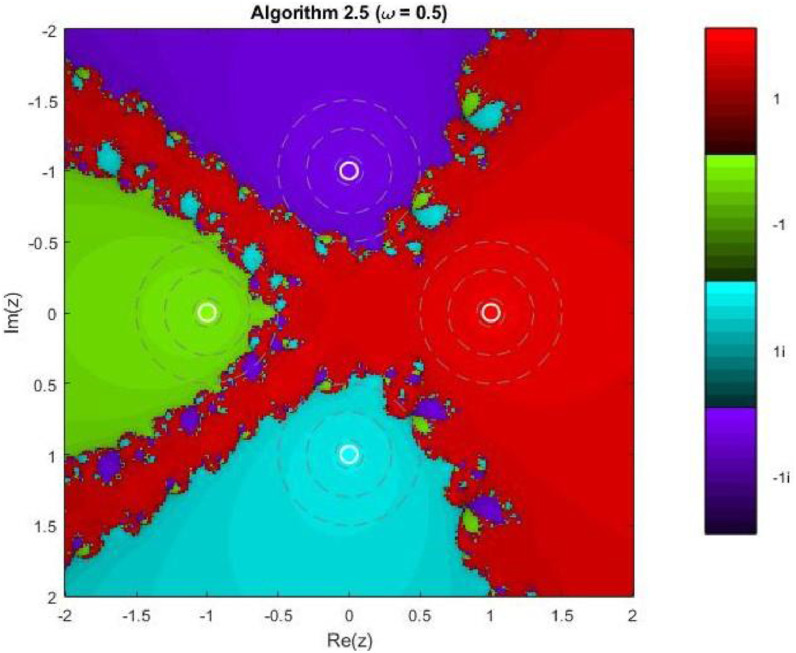
Fractal for Algorithm 2.5.

**Fig. 21:**
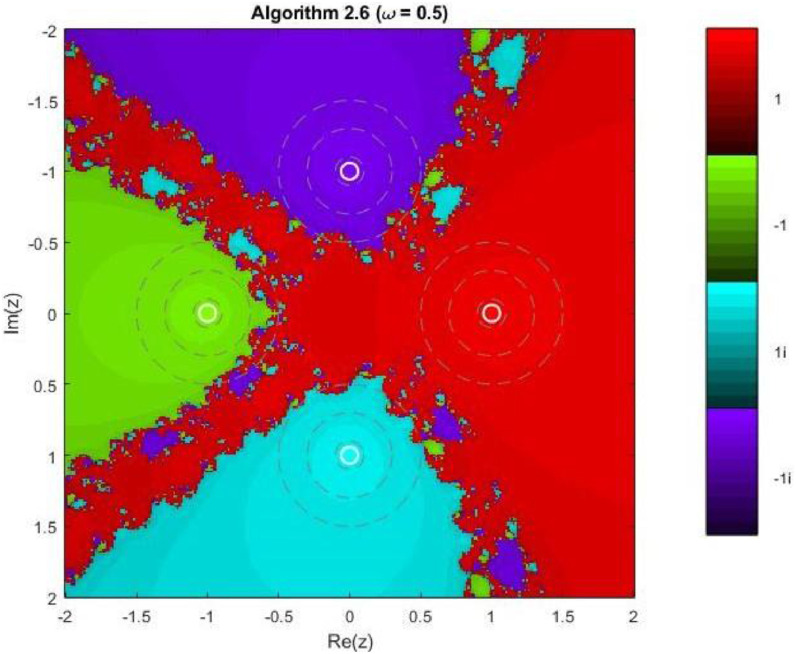
Fractal for Algorithm 2.6.

**Fig. 22:**
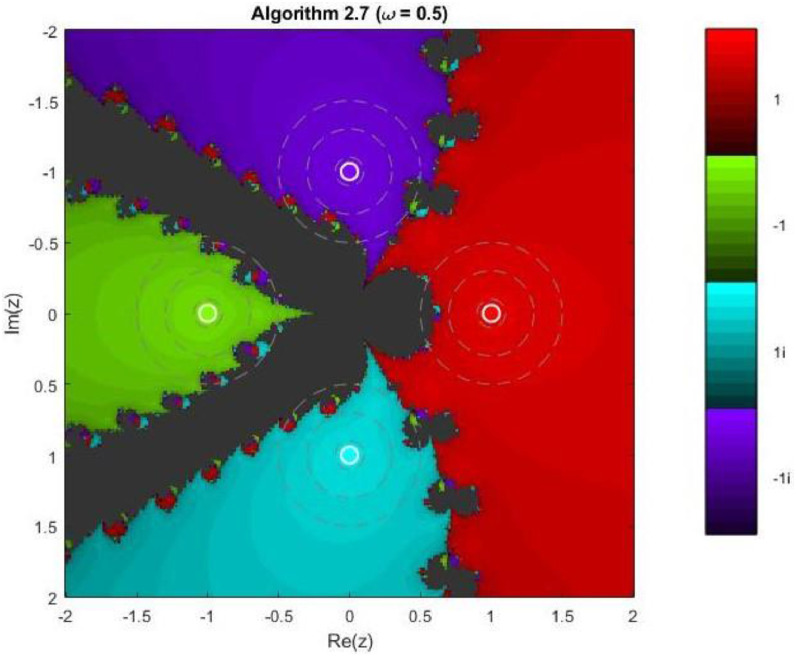
Fractal for Algorithm 2.7.

**Fig. 23:**
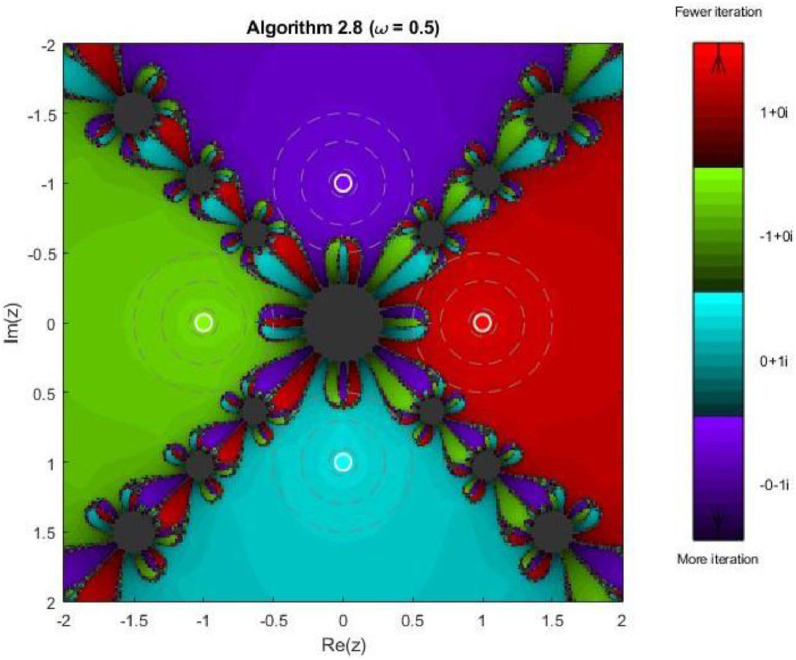
Fractal for Algorithm 2.8.

**Fig. 24:**
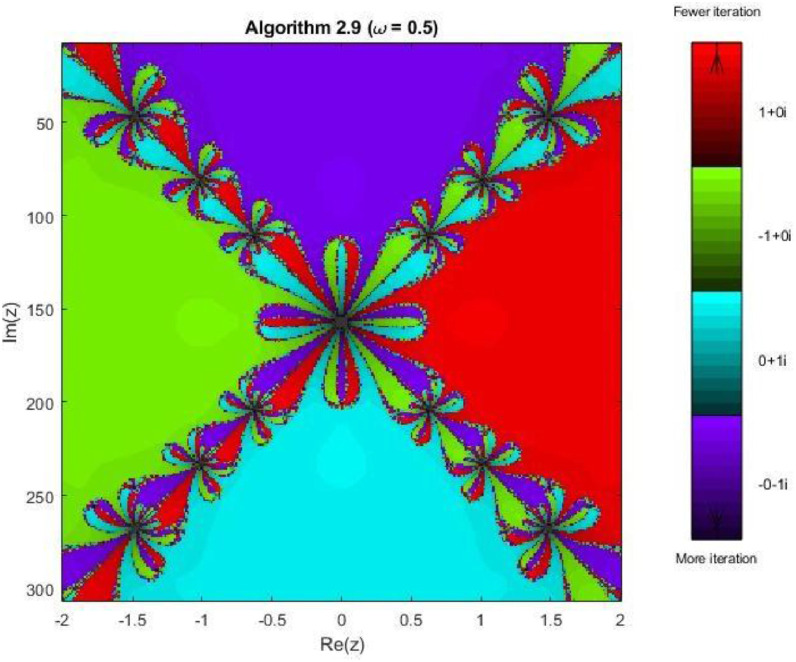
Fractal for Algorithm 2.9.

Example 5 Another test function, 4th degree polynomial, is compiled for the performance and robustness of the proposed iterative schemes. Fractal plots are generated for a biquadratic polynomial using Algorithms 2.4 to 2.9, as shown in Fig. 19, Fig. 20, Fig. 21, Fig. 22, Fig. 23, Fig. 24. The biquadratic equation possesses four distinct roots, leading to four principal basins of attraction in the complex plane. Distinct colours and the brightness of each colour implies the same as in previous plots.

Here in first plot of this group, illustrates the fractal structure obtained using Algorithm 2.4. The four basins of attraction are clearly identifiable. However, the boundaries separating them are highly irregular and densely interwoven. These intricate fractal boundaries indicate strong sensitivity to initial conditions, particularly in regions where multiple basins intersect. Although convergence near the roots is relatively rapid, the algorithm exhibits slower convergence along the basin boundaries. As shown in Fig. 20, Algorithm 2.5 provides a modest improvement in basin regularity. The attraction regions become more symmetric, and the chaotic boundary zones are slightly reduced compared to Algorithm 2.4. Nevertheless, noticeable oscillatory patterns persist near the separatrix regions, indicating that the method remains sensitive to initial guesses in transition areas. The next Fig. 21 demonstrates that Algorithm 2.6 yields further refinement of the fractal geometry. The basins of attraction expand more uniformly, and the boundary regions become thinner and more structured. The color intensity suggests a reduction in the average number of iterations required for convergence, reflecting improved efficiency and numerical stability.

In Fig. 22, Algorithm 2.7 exhibits a distinctive convergence pattern. While the four primary basins remain dominant, larger dark regions appear near the central intersection of the basins, indicating slower convergence or delayed stabilization for certain initial points. This behavior highlights a trade-off between rapid convergence near the roots and increased sensitivity in regions where multiple attraction domains compete. Fig. 23 reveals highly symmetric and elaborate fractal structures produced by Algorithm 2.8. The basin boundaries display petal-like patterns and rotational symmetry, characteristic of higher-order iterative schemes. Despite the increased geometric complexity, convergence within each basin remains fast, and the basins are well separated, demonstrating strong local convergence properties.

Last plot this group, Fig. 24, Algorithm 2.9 achieves the most stable and uniform convergence behavior among all tested methods. The basins of attraction are smoother and more extensive, with sharply defined boundaries and minimal regions of slow convergence. The relatively uniform color intensity across each basin confirms faster and more consistent convergence throughout the complex plane. On the whole, the fractal analysis for the biquadratic polynomial confirms a clear enhancement in convergence performance from Algorithm 2.4 to Algorithm 2.9. Lower-order schemes exhibit highly complex and fragmented basin boundaries, whereas higher-order methods—particularly Algorithms 2.8 and 2.9 produce smoother, more symmetric basins with reduced sensitivity to initial guesses. These observations demonstrate that the proposed higher-order algorithms retain their effectiveness even for equations with multiple interacting roots, reinforcing their reliability and robustness for solving nonlinear problems of increased algebraic complexity.

## Conclusion

In this study, we have proposed novel quadrature-based iterative methods for the efficient computation of simple roots of nonlinear equations. The proposed schemes attain high order convergence and demonstrate improved stability and convergence efficiency compared with classical methods, as evidenced by the fractal basin-of-attraction analysis. In addition to their numerical performance, these methods offer valuable insight into the dynamical behaviour of iterative processes. It provides a robust and reliable framework with potential applications in mathematics, physics, engineering, and other applied sciences. Implications for future research several interesting lines of future investigation are suggested by this study. Naturally, one would like to extend these approaches from single equations to a system of nonlinear equations where the multiplicity is unknown in advance. It would be of interest as well to construct optimally efficient higher order methods that combine the best aspects of the current approach. The flexibility and stability of the methods could be further improved if we take into consideration some fractional calculus or adaptive error control mechanism, respectively.

## Limitations

Like all iterative methods for the implementation of Algorithms 2.4, 2.5, 2.6, 2.7, 2.8 and 2.9 have some of the limitations:

• Smoothness: The nonlinear function should be continuous and differentiable near the root.
• Numerical stability: If the denominator of the corrector function approaches zero, the method may fail to converge.
• Function behavior: Rapid changes or oscillations in the function can hinder convergence of numerical methods.

## Ethics statements

*Not Applicable.*

## CRediT authorship contribution statement

**Farooq Ahmed Shah:** Conceptualization, Methodology, Supervision. **Syeda Rameesha Hamdani:** Writing – original draft, Investigation, Validation. **Fikadu Tesgera Tolasa:** Investigation, Validation. **Iftikhar Haider:** Methodology, Writing – review & editing.

## Declaration of competing interest

The authors declare that they have no known competing financial interests or personal relationships that could have appeared to influence the work reported in this paper.

## Data Availability

No data was used for the research described in the article.

## Appendix Supplementary materials

Image, application 1
